# Force-induced Caspase-1-dependent pyroptosis regulates orthodontic tooth movement

**DOI:** 10.1038/s41368-023-00268-7

**Published:** 2024-01-15

**Authors:** Liyuan Chen, Huajie Yu, Zixin Li, Yu Wang, Shanshan Jin, Min Yu, Lisha Zhu, Chengye Ding, Xiaolan Wu, Tianhao Wu, Chunlei Xun, Yanheng Zhou, Danqing He, Yan Liu

**Affiliations:** 1 Department of Orthodontics, Central Laboratory, Peking University School and Hospital for Stomatology & National Center for Stomatology & National Clinical Research Center for Oral Diseases & National Engineering Research Center of Oral Biomaterials and Digital Medical Devices & Beijing Key Laboratory of Digital Stomatology & Research Center of Engineering and Technology for Computerized Dentistry Ministry of Health & NMPA Key Laboratory for Dental Materials & National Engineering Research Center of Oral Biomaterials and Digital Medical Devices, Beijing, China; 2 Peking University Hospital of Stomatology Fourth Division, Beijing, China

**Keywords:** Cell death, Mesenchymal stem cells, Bone remodelling

## Abstract

Pyroptosis, an inflammatory caspase-dependent programmed cell death, plays a vital role in maintaining tissue homeostasis and activating inflammatory responses. Orthodontic tooth movement (OTM) is an aseptic force-induced inflammatory bone remodeling process mediated by the activation of periodontal ligament (PDL) progenitor cells. However, whether and how force induces PDL progenitor cell pyroptosis, thereby influencing OTM and alveolar bone remodeling remains unknown. In this study, we found that mechanical force induced the expression of pyroptosis-related markers in rat OTM and alveolar bone remodeling process. Blocking or enhancing pyroptosis level could suppress or promote OTM and alveolar bone remodeling respectively. Using *Caspase-1*^−/−^ mice, we further demonstrated that the functional role of the force-induced pyroptosis in PDL progenitor cells depended on Caspase-1. Moreover, mechanical force could also induce pyroptosis in human ex-vivo force-treated PDL progenitor cells and in compressive force-loaded PDL progenitor cells in vitro, which influenced osteoclastogenesis. Mechanistically, transient receptor potential subfamily V member 4 signaling was involved in force-induced Caspase-1-dependent pyroptosis in PDL progenitor cells. Overall, this study suggested a novel mechanism contributing to the modulation of osteoclastogenesis and alveolar bone remodeling under mechanical stimuli, indicating a promising approach to accelerate OTM by targeting Caspase-1.

## Introduction

Pyroptosis is a lytic type of programmed cell death, which is initiated by inflammatory caspases and characterized by gasdermin (GSDM)-mediated membrane pore-formation and the release of cellular contents^1,2^ Pyroptosis could be activated by extracellular or intracellular stimulation, including pathogen infection, inflammation, tumorigenesis, and mechanical forces, which play an important role in maintaining tissue homeostasis and activating the inflammatory responses.^3,4^ Depending on different environmental stimuli and inflammatory caspases, pyroptosis can be divided into canonical and non-canonical types.^5^ In canonical pyroptosis, inflammasomes such as Nod-like receptor protein 3 (NLRP3) activate Caspase-1 to cleave gasdermin D (GSDMD) and process pro-IL-1β into mature IL-1β.^1^ In non-canonical pyroptosis, Caspase-11/4/5 is activated to cleave GSDMD upon recognition of cytosolic lipopolysaccharide, which is independent of inflammasomes and Caspase-1.^2^

Orthodontic tooth movement (OTM) is an aseptic inflammatory bone remodeling process induced by mechanical force stimulation.^6^ Under force stimulation, numerous inflammatory cytokines, chemokines, and increased activation of immune cells were detected in periodontal tissues.^7–9^ Periodontal ligament (PDL) stem/progenitor cells were the main cellular components in the periodontal tissues, constantly receive force stimuli and contribute to the inflammatory responses and bone remodeling process during OTM.^10^ Our previous studies have reported that the expressions of inflammatory cytokines, chemokines, and gas molecules such as hydrogen sulfide were all increased in the force-stimulated PDL stem/progenitor cells.^11–13^ In addition, cyclic stretch could activate NLRP inflammasomes and induce the release of IL-1β via a Caspase-1-related mechanism in PDL cells in vitro.^14^ However, whether and how mechanical force induces PDL stem/progenitor cell pyroptosis and thus influences OTM and alveolar bone remodeling remain unknown.

Transient receptor potential (TRP) calcium channel is a classic mechanosensitive channel contributing to the transduction of mechano-signals into biological responses in various tissues and cells.^15^ TRP subfamily V member 4 (TRPV4) could regulate mechano-transduction, inflammation activation, and mechanical force-induced alveolar bone remodeling.^16^ Previously, we have found that TRPV4 was involved in the modulation of PDL stem cell function during OTM both in vivo and in vitro.^17^ In addition, a previous study also suggested that TRPV4 could mediate airway epithelial cell pyroptosis in chronic obstructive pulmonary disease.^18^ Therefore, we hypothesize that TRPV4 participates in force-induced pyroptosis in PDL progenitor cells.

In the present study, we aim to illustrate whether and how mechanical force induced PDL progenitor cell pyroptosis and influenced OTM and alveolar bone remodeling. By using OTM animal models, force-induced human PDL progenitor cells ex-vivo, and a compressive force loading model in vitro, we found that mechanical force induced Caspase-1-dependent pyroptosis in PDL progenitor cells, which contributed to OTM and alveolar bone remodeling. This study shed light on a novel mechanism of OTM and indicated that targeting Caspase-1 might be a promising approach to accelerate OTM.

## Results

### Force induces PDL progenitor cell pyroptosis during OTM and alveolar bone remodeling in vivo

**Fig. 1:**
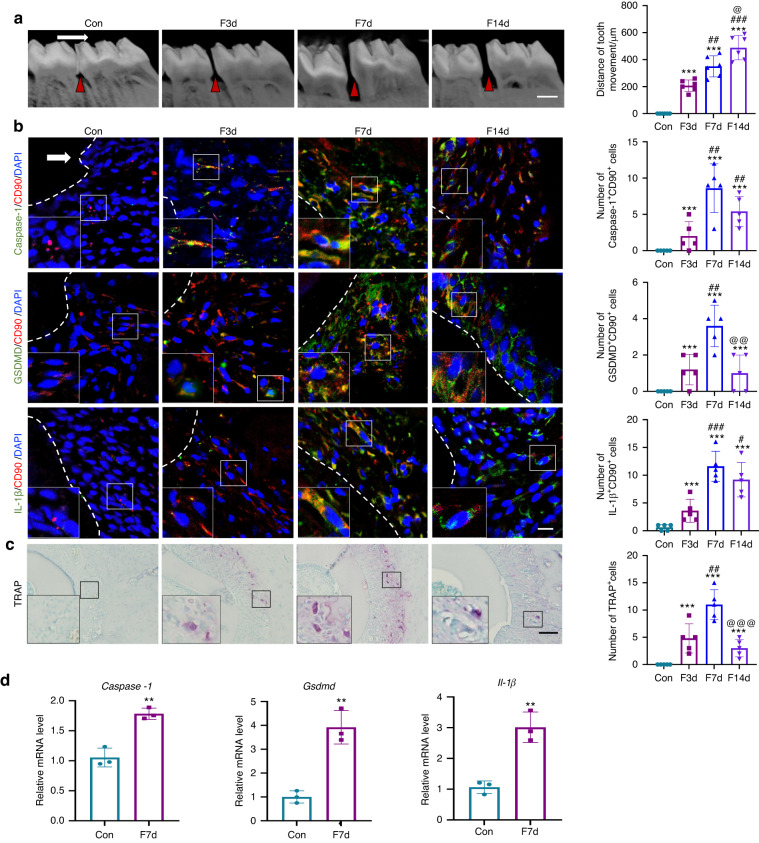
Mechanical force induces pyroptosis during OTM and alveolar bone remodeling in vivo. **a** Representative image of micro-CT and semiquantification analysis of force-induced OTM distance in rats. Scale bar: 1 mm. **b** Representative immunofluorescence images on the compression side of distobuccal roots and semiquantification analysis of double-labeled cells. Dashed lines mark the outline of distobuccal roots. Scale bar: 50 µm. **c** Representative tartrate-resistant acid phosphatase (TRAP) staining images of distobuccal roots. Scale bar: 200 µm. Results were presented as mean ± SD. *n* = 5 biologically independent samples. **d** Real time-PCR of *Caspase-1, Il-1β*, and *Gsdmd* in periodontal tissues. Results were presented as mean ± SD. *n* = 3 biologically independent samples. **P < 0.01, ***P < 0.001 versus Con; #*P* < 0.05, ##*P* < 0.01, ###*P* < 0.001 versus F3d; @*P* < 0.05, @@*P* < 0.01, @@@*P* < 0.001 versus F7d. The white arrow represents the direction of the force application. Large boxed areas show high magnification views of the small boxed areas

To investigate whether mechanical force-induced pyroptosis regulates alveolar bone remodeling in vivo, a classic force-induced OTM and alveolar bone remodeling model was established. Micro-CT images showed that the OTM distance in rats gradually increased to (207 ± 17.64) µm, (350 ± 31.62) µm, and (488 ± 36.64) µm after force loading for 3 d, 7 d, and 14 d (Fig. 1a). CD90 has been widely used as a marker for characterizing PDL progenitor cells in rats (Kon et al. 2009; Hosoya et al. 2012), as it is expressed in stem/progenitor cells (Dennis et al. 2007). Immunofluorescence showed that the number of Caspase-1^+^CD90^+^ cells, GSDMD^+^CD90^+^ cells, and IL-1β^+^CD90^+^ cells was all increased in the compression side of the periodontal tissues after force loading for 3 d and lasted to 14 d, while force loading for 7 d triggered the strongest responses (Fig. 1b and Supplementary Fig. S1). The number of tartrate-resistant acid phosphatase (TRAP)^+^ osteoclasts showed a similar trend (Fig. 1c). However, on the tension side, the expressions of pyroptosis-related markers did not change compared with the control group (Supplementary Fig. S2). Moreover, after force stimulation for 7 d, the periodontal tissues from the mesial side of the first molars were collected and the expression of pyroptosis-related genes including *Caspase-1*, *Gsdmd* and *IL-1β* were significantly upregulated (Fig. 1d).

### Force-induced pyroptosis modulates OTM and alveolar bone remodeling in a Caspase-1 depended manner

**Fig. 2:**
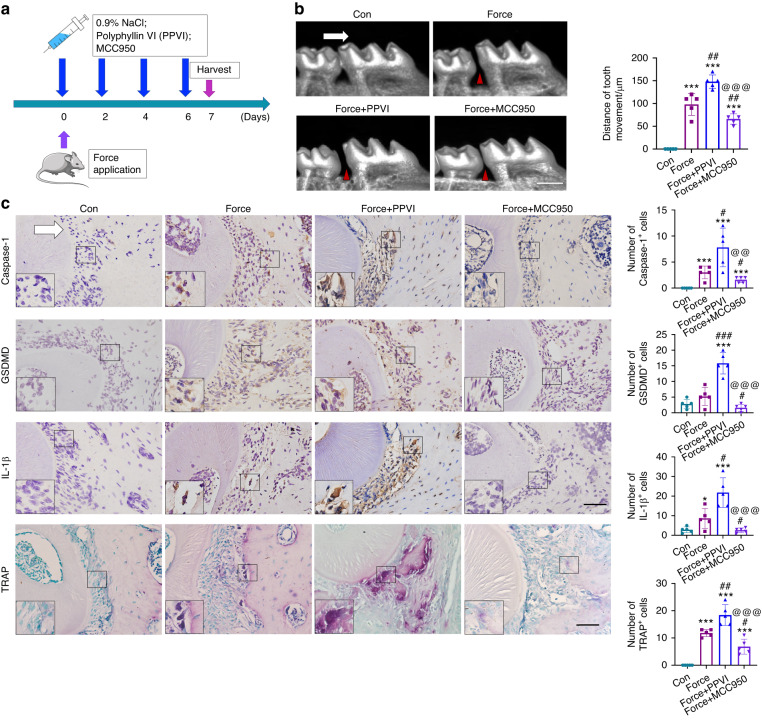
Modulating pyroptosis level influences OTM and alveolar bone remodeling. **a** Schematic of the in vivo study. **b** Representative micro-CT images of OTM in mice. Con: mice without force stimuli in vivo; Force: mice receiving force stimuli for 7 d; Force+Polyphyllin VI (PPVI): mice receiving force stimuli for 7 d and the application of pyroptosis activator PPVI treatment. Force+MCC950: mice receiving force stimuli for 7 d and the application of pyroptosis inhibitor MCC950 treatment. Scale bar: 500 µm. **c** Representative immunohistochemical staining images and TRAP staining images of the compression side of distobuccal roots. The expressions of pyroptosis-related proteins Caspase-1, GSDMD, IL-1β, and the TRAP positive cells were detected. Large boxed areas show high magnification views of the small boxed areas. Scale bar: 100 µm. *P < 0.05, ***P < 0.001 versus Con; #*P* < 0.05, ##*P* < 0.01, ###*P* < 0.001 versus Force; @@*P* < 0.01, @@@*P* < 0.001 versus Force+PPVI. Results were presented as mean ± SD. *n* = 5 biologically independent samples. The white arrow represents the direction of the force application

To further explore the influence of pyroptosis level on OTM, we enhanced or blocked the pyroptosis level by systemic administration of the pyroptosis activator Polyphyllin VI (PPVI) or inhibitor MCC950 in mice respectively (Fig. 2a). After force loading for 7 d, the OTM distance was increased after PPVI injection and decreased after MCC950 injection compared with the force group (Fig. 2b). Concomitantly, after PPVI injection, the force-induced expressions of Caspase-1, GSDMD and IL-1β were further elevated in the periodontal tissues, whereas the MCC950 injection partially reversed the expressions of pyroptosis-related markers compared with the force group. Moreover, the number of TRAP^+^ osteoclasts increased after force application, which was further enhanced by the PPVI injection and suppressed by the MCC950 injection (Fig. 2c).

**Fig. 3:**
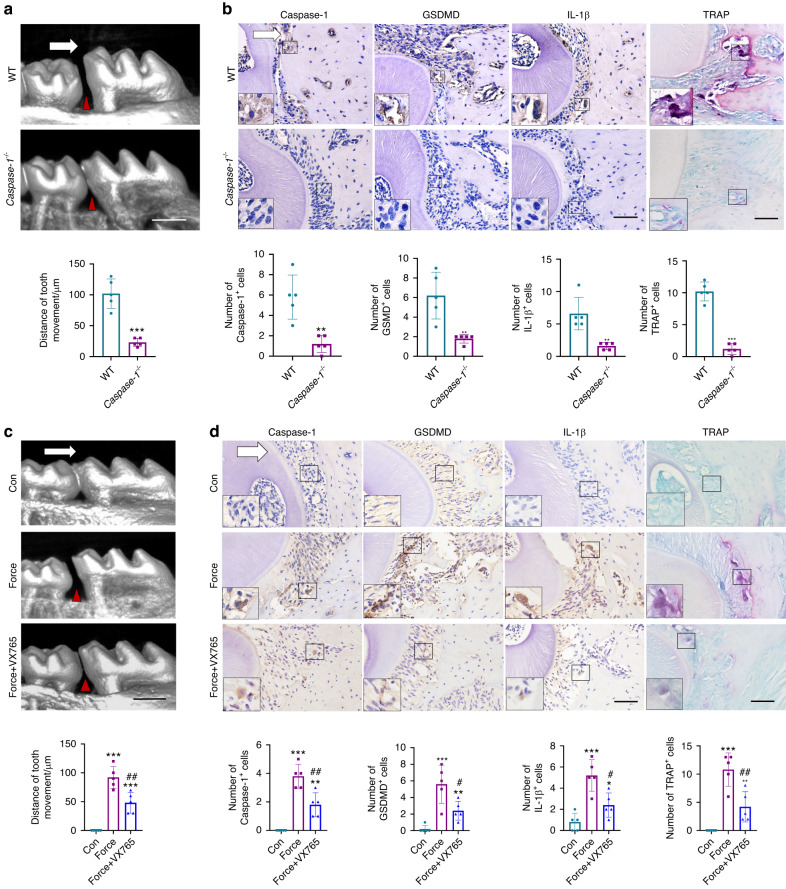
Force-induced pyroptosis modulates OTM and alveolar bone remodeling in a Caspase-1-depended manner. **a** Representative micro-CT images of force-induced tooth movement distance in wild type (WT) or *Caspase-1*^−/−^ mice. Scale bar: 500 µm. **b** Representative immunohistochemical staining images and TRAP staining images of the compression side of distobuccal roots. Large boxed areas show high magnification views of the small boxed areas. Scale bars: 100 µm. **P < 0.01, ***P < 0.001 versus WT. **c** Representative images of micro-CT of force-induced tooth movement distance in mice with or without Caspase-1 inhibitor Belnacasan (VX765) application. Scale bar: 500 µm. **d** Representative immunohistochemically stained images and TRAP staining images of the compression sides of distobuccal roots. Large boxed areas show high magnification views of the small boxed areas. Scale bars: 100 µm. *P < 0.05, **P < 0.01, ***P < 0.001 versus Con; #*P* < 0.05, ##*P* < 0.01 versus Force. The white arrow represents the direction of the force application. Results were presented as mean ± SD. *n* = 5 biologically independent samples

Caspase-1 was a key factor to cleave GSDMD in canonical pyroptosis, therefore we further confirm whether force-induced pyroptosis requires the activation of Caspase-1 using *Caspase-1*^−/−^ mice. After force application for 7d, the OTM distance was significantly reduced in *Caspase-1*^−/−^ mice (Fig. 3a). Correspondingly, the expressions of Caspase-1, GSDMD, IL-1β, as well as the number of TRAP^+^ osteoclasts were all significantly decreased in the periodontal tissues of *Caspase-1*^−/−^ mice compared with WT mice (Fig. 3b). These data suggest that mechanical force could induce Caspase-1-dependent pyroptosis, which further contributed to the OTM and alveolar bone remodeling.

In addition, the Caspase-1 inhibitor Belnacasan (VX765) was also injected into mice every other day during the force-induced tooth movement process. After VX765 injection, the tooth movement distance decreased significantly compared to the Force group (Fig. 3c). Moreover, the force-induced upregulated expressions of Caspase-1, GSDMD, and IL-1β were partially reversed, as well as the number of TRAP^+^ osteoclasts (Fig. 3d).

### Mechanical force induces pyroptosis in human PDL progenitor cells and influences osteoclastic activity

**Fig. 4:**
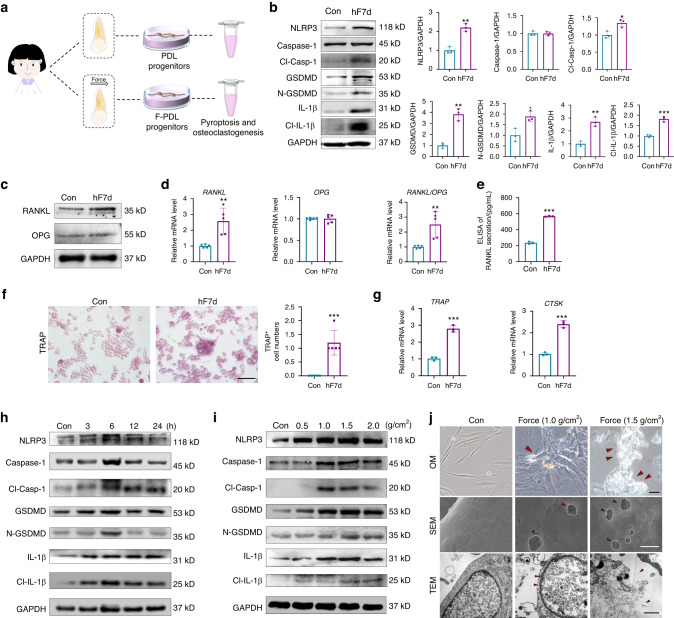
Mechanical force induces pyroptosis in human PDL progenitor cells and influences osteoclastic activity. **a** Schematic of the isolation of ex vivo human PDL (h-PDL) progenitor cells. **b** Western blotting of pyroptosis-related proteins in ex-vivo h-PDL progenitor cells under clinical force for 7 d. **c**, **d** Western blotting and Real time-PCR of RANKL and OPG expressions in ex-vivo h-PDL progenitor cells. **e** ELISA of RANKL secretion in ex-vivo h-PDL progenitor cells. **f** Representative images of TRAP staining of osteoclasts in peripheral blood mononuclear cells (PBMCs) co-cultured with ex-vivo h-PDL progenitor cells. Scale bar: 50 µm. **g** Real time-PCR of *TRAP* and *Cathepsin K* (*CTSK*)expressions in PBMCs. *P < 0.05, **P < 0.01, ***P < 0.001 versus Con. **h**, **i** Western blotting of pyroptosis-related proteins in PDL progenitor cells under 1.5 g/cm^2^ compressive force at different time points and under different force loading for 6 h in vitro. **j** Representative images of optical microscope (OM), scanning electron microscope (SEM) and transmission electron microscope (TEM) of PDL progenitor cells under 1.0 g/cm^2^ and 1.5 g/cm^2^ compressive force loading for 6 h in vitro. The red arrows in OM show the bubbles. Scale bar: 20 µm. The white circles in SEM show multiple pores in the membranes, and the red arrows in TEM show membrane disruption, cell swelling, and lysis. Scale bar: 1 µm. Results were presented as mean ± SD. *n* = 3–5 biologically independent samples

PDL stem/progenitor cells are the main cells responding to mechanical force and contributing to OTM, therefore we further detect whether mechanical force induces pyroptosis in PDL progenitor cells under force stimuli. Firstly, the expression of pyroptosis-related markers in ex-vivo h-PDL progenitor cells isolated from the same patients with or without force loading were detected (Fig. 4a). The protein expression of pyroptosis-related markers including NLRP3 inflammasomes, cleaved Caspase-1 (Cl-Casp-1), GSDMD, cleaved GSDMD (N-GSDMD), as well as IL-1β and cleaved IL-1β (Cl-IL-1β), were all significantly increased in h-PDL progenitor cells with force application for 7 d (hF7d group) (Fig. 4b). The mRNA expressions of pyroptosis-related genes showed the same trend (Supplementary Fig. S3a).

To verify the relationship between PDL progenitor pyroptosis and osteoclastic activity, ex-vivo h-PDL progenitor cells with or without orthodontic force pretreatment were co-cultured with peripheral blood mononuclear cells (PBMCs). The protein expression of RANKL significantly enhanced in the hF7d group, whereas OPG expression remained unchanged (Fig. 4c and Supplementary Fig. S3b). Correspondingly, the gene expressions of *RANKL* and *RANKL/OPG* ratio were upregulated in the hF7d group (Fig. 4d). Moreover, the secretion of RANKL also increased detected by ELISA (Fig. 4e). In addition, the number of TRAP^+^ osteoclasts increased significantly in the hF7d group (Fig. 4f), and the mRNA expression of *Cathepsin K* (*CTSK*)) and *TRAP* also increased significantly in osteoclasts (Fig. 4g). These data indicated that mechanical force induced pyroptosis in human ex-vivo PDL progenitor cells and influenced osteoclastic activity.

In addition, compressive force was further applied to PDL progenitor cells in vitro. Western blotting revealed that under 1.5 g/cm^2^ force stimuli, the expression of pyroptosis-related proteins increased from 3 h and lasted to 24 h, which reached to the peak at 6 h (Fig. 4h, and Supplementary Fig. S4a). In addition, under different force stimuli for 6 h, the protein expression of pyroptosis-related markers increased from 0.5 g/cm^2^ and reached to the peak at 1.5 g/cm^2^ or 2.0 g/cm^2^. (Fig. 4i and Supplementary Fig. S4b). Correspondingly, real-time PCR showed the similar trends (Supplementary Fig. S4c, d). Notably, no significant change in the Caspase-5 expression was detected after force stimulation, indicating that force induced the Caspase-1-dependent canonical type of pyroptosis instead of the noncanonical type (Supplementary Fig. S4f).

The pyroptotic morphology of swollen and flat cells with blurred cellular contour and large bubbles were observed in optical microscope (OM) images. Moreover, scanning electron microscope (SEM) and transmission electron microscope (TEM) images showed multiple pores in the membranes of PDL progenitor cells under 1.0 g/cm^2^ force stimulation, and more obvious membrane disruption, cell swelling, and lysis were observed under 1.5 g/cm^2^ force stimulation (Fig. 4j and Supplementary Fig. S4e). Overall, these findings revealed that mechanical force induced pyroptosis in PDL progenitor cells both in vivo and in vitro.

### Regulation of PDL progenitor cell pyroptosis influences osteoclastic activity

**Fig. 5:**
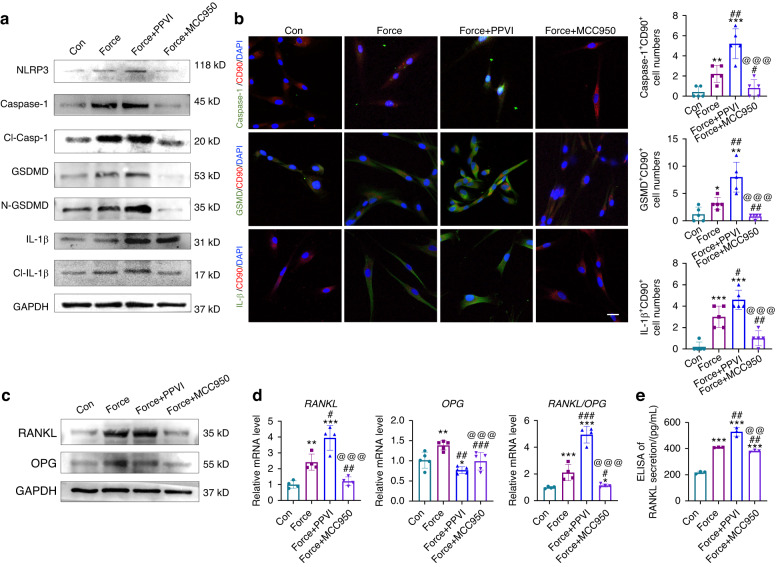
Regulation of PDL progenitor pyroptosis influences osteoclastic activity. **a** Western blotting of pyroptosis-related proteins in PDL progenitor cells under 1.5 g/cm^2^ force loading for 6 h with or without the appliance of PPVI and MCC950 in vitro. Con: PDL progenitor cells without force loading and drug application; Force: PDL progenitor cells under 1.5 g/cm^2^ force loading for 6 h; Force + PPVI: PDL progenitor cells with force loading and pyroptosis activator PPVI treatment. Force + MCC950: PDL progenitor cells with force loading and pyroptosis inhibitor MCC950 treatment. **b** Representative immunocytofluorescense images of PDL progenitor cells of Con, Force, Force + PPVI and Force + MCC950. Scale bar: 10 µm. *n* = 5 independent experiments. **c** Western blotting of RANKL and OPG in PDL progenitor cells of Con, Force, Force + PPVI and Force + MCC950. **d** Real time-PCR of *RANKL, OPG*, and the ratio of *RANKL/OPG* in PDL progenitor cells of Con, Force, Force + PPVI and Force + MCC950. **e** ELISA of RANKL secretion in PDL progenitor cells of Con, Force, Force + PPVI and Force + MCC950. *P < 0.05, **P < 0.01, ***P < 0.001 versus Con; #*P* < 0.05, ##*P* < 0.01, ###*P* < 0.001 versus Force; @*P* < 0.05, @@*P* < 0.01, @@@*P* < 0.001 versus Force+PPVI. Results were presented as mean ± SD. *n* = 3–5 biologically independent samples

Pyroptosis activator PPVI and inhibitor MCC950 were also utilized to treat force-loaded PDL progenitor cells in vitro. Western blotting analysis revealed that force increased the expression level of pyroptosis-related proteins, including NLRP3, Caspase-1, Cl-Casp-1, and the downstream GSDMD, N-GSDMD, IL-1β, and Cl-IL-1β. These protein expression levels were further enhanced after PPVI application and partially suppressed after MCC950 application (Fig. 5a and Supplementary Fig. S5a). Moreover, the immunofluorescence images also showed that the numbers of GSDMD^+^CD90^+^ cells, Caspase-1^+^CD90^+^ cells, and IL-1β^+^CD90^+^ cells were all increased after the PPVI application and decreased after the MCC950 application compared to the Force group (Fig. 5b). Furthermore, the ratio of RANKL/OPG was upregulated after PPVI application and downregulated after MCC950 application compared to the Force group (Fig. 5c and Supplementary Fig. S5b). Real-time PCR also showed the same trend (Fig. 5d). Moreover, ELISA showed that the secretion of RANKL increased after PPVI application and decreased after MCC950 application compared with the force group (Fig. 5e).

**Fig. 6:**
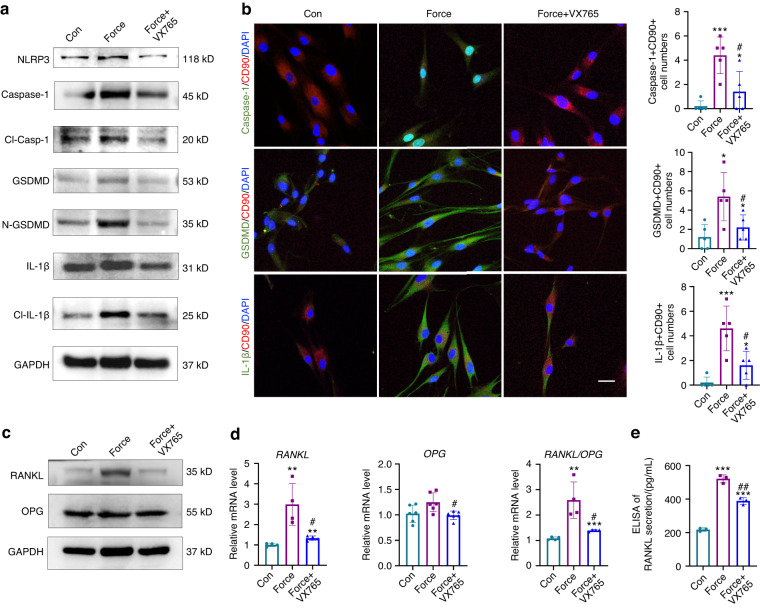
Regulation of Caspase-1 influences RANKL/OPG expression in PDL progenitor cells in vitro. **a** Western blotting of pyroptosis-related proteins in PDL progenitor cells under 1.5 g/cm^2^ force loading for 6 h with or without the appliance of the Caspase-1 inhibitor VX765. Con: PDL progenitor cells without force loading and drug application; Force: PDL progenitor cells under 1.5 g/cm^2^ force loading for 6 h; Force + VX765: PDL progenitor cells with force loading and VX765 treatment. **b** Representative immunocytofluorescense images of PDL progenitor cells under force loading with or without VX765 application. Scale bar:10 µm. *n* = 5 independent experiments. **c** Western blotting of RANKL and OPG in PDL progenitor cells under force loading with or without VX765 application. **d** Real time-PCR of *RANKL, OPG*, and the ratio of *RANKL/OPG* under 1.5 g/cm^2^ force loading for 6 h. **e** ELISA of RANKL secretion in PDL progenitor cells of Con, Force, Force+VX765. *P < 0.05, **P < 0.01, ***P < 0.001 versus Con; #*P* < 0.05, ##*P* < 0.01, ###*P* < 0.001 versus Force. Results were presented as mean ± SD. *n* = 3–5 biologically independent samples

In addition, the Caspase-1 inhibitor Belnacasan (VX765) was further utilized to treat force-loaded PDL progenitor cells in vitro. VX765 application reduced the force-induced pyroptosis-related protein expressions of NLRP3, Caspase-1, Cl-Casp-1, GSDMD, N-GSDMD, IL-1β, and Cl-IL-1β compared to the Force group (Fig. 6a and Supplementary Fig. S6a). In addition, the immunofluorescence images also showed that the numbers of Caspase-1^+^CD90^+^ cells, GSDMD^+^CD90^+^ cells, and IL-1β^+^CD90^+^ cells were all decreased after VX765 application compared to the Force group (Fig. 6b). Moreover, the application of VX765 significantly reversed the upregulated protein expression of RANKL and RANKL/OPG ratio (Fig. 6c and Supplementary Fig. S6b). Similar results were found in their gene expression levels (Fig. 6d). Moreover, the secretion of RANKL was also decreased after VX765 application compared with the force group by ELISA (Fig. 6e). Taken together, these data suggest that force-induced pyroptosis in PDL progenitor cells required the activation of Caspase-1, which further contributed to the osteoclastogenesis.

### TRPV4 signaling is involved in force-induced pyroptosis in PDL progenitor cells

**Fig. 7:**
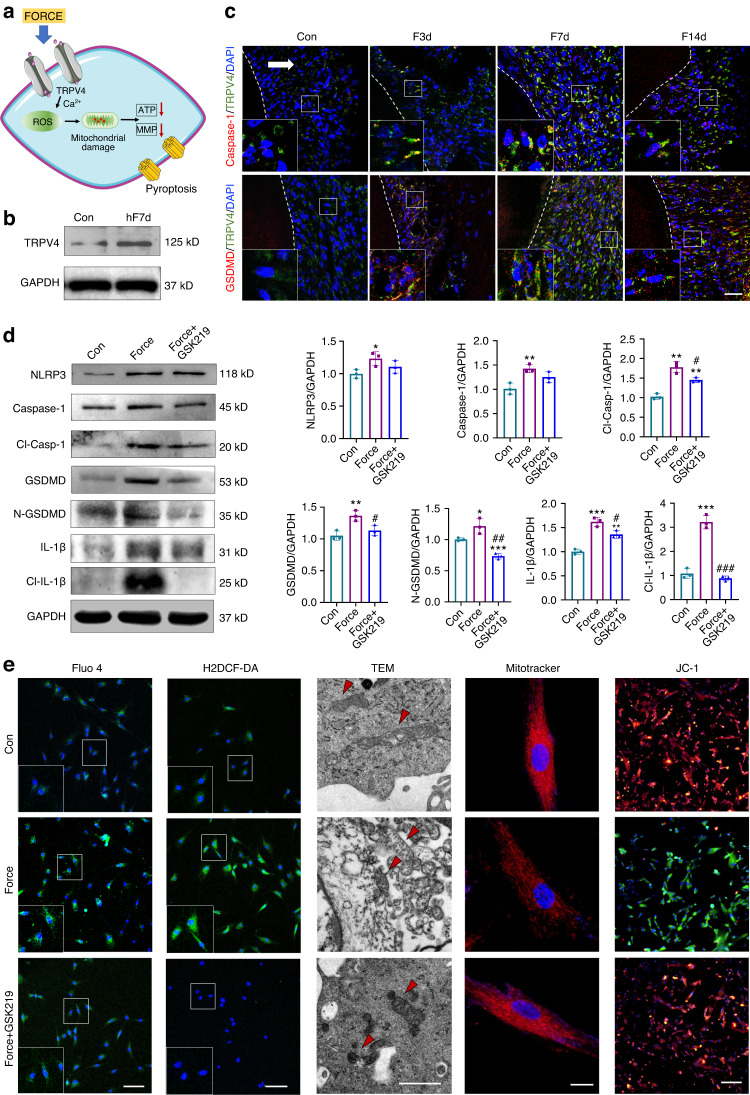
TRPV4 signaling is involved in force-induced pyroptosis in PDL progenitor cells. **a** Schematic of the experiment. **b** Western blotting of TRPV4 in ex-vivo h-PDL progenitor cells with or without clinical orthodontic force stimulated for 7 d. **c** Representative immunofluorescence images on the compression side of distobuccal roots. Dashed lines mark the outline of roots. Arrow represents the direction of the force. Scale bar: 50 µm. **d** Western blotting of pyroptosis-related proteins and semiquantification analysis in PDL progenitor cells under 1.5 g/cm^2^ force loading for 6 h. *P < 0.05, **P < 0.01, ***P < 0.001 versus Con, #*P* < 0.05, ##*P* < 0.01, ###*P* < 0.001 versus Force. **e** Representative immunofluorescence images of Fluo-4 (green) in PDL progenitor cells. Scale bar: 100 µm. Representative photomicrographs of intracellular reactive oxygen species (ROS) shown by H2DCF-DA (green) in PDL progenitor cells. Scale bar: 50 µm. Representative images of mitochondrial morphology detected by TEM (Scale bar: 1 µm). Representative images of mitochondrial morphology detected by MitoTracker Red (Scale bar: 5 µm). Representative images of PDL progenitor cells mitochondrial membrane potential detected by JC-1. Scale bar: 100 µm

TRPV channels could induce biological cellular responses under mechanical stimulation. Western blotting and immunofluorescence staining showed that the expression of TRPV4 was enhanced in ex-vivo h-PDL progenitor cells in the hF7d group (Fig. 7b). In rat OTM models, the number of Caspase-1^+^TRPV4^+^ cells and GSDMD^+^TRPV4^+^ cells increased from F3d to F7d and F14d (Fig. 7c and Supplementary Fig. S7). In addition, real-time PCR of the rat periodontal tissues after force application for 7 d showed that *Trpv4* increased significantly, whereas no significant difference was detected on *Trpv1-3* (Supplementary Fig. S8a). Moreover, force-induced increased expression of pyroptosis-related markers was partially suppressed after application of a TRPV4 inhibitor GSK2193874 (GSK219) (Fig. 7d).

TRPV4 regulates numerous cellular functions through intracellular Ca^2+^ influx. Therefore, we hypothesized that TRPV4 regulates PDL progenitor cell pyroptosis through Ca^2+^ influx, which further induces reactive oxygen species (ROS) elevation and mitochondrial damage. Immunofluorescence staining of Fluo 4 and H2DCF-DA showed that force increased Ca^2+^ influx and intracellular ROS in PDL progenitor cells, which were blocked by the application of GSK219 (Fig. 7e). TEM and mito-tracker dyes showed that mitochondria were swollen and fragmented in force-treated PDL progenitor cells. After GSK219 application, mitochondrial morphology tended to be normal (Fig. 7e). The functional consequences of force-induced morphological changes in the mitochondria including decreased mitochondrial membrane potential detected by JC-1 and impaired ATP production were reversed after GSK219 application (Fig. 7e, and Supplementary Fig. S8b, c). In sum, these findings demonstrated that TRPV4 signaling plays an important role in regulating force-induced Caspase-1-dependent pyroptosis in PDL progenitor cells (Fig. 7a).

## Discussion

Pyroptosis plays a vital role in activating inflammatory responses under mechanical stimuli. However, whether and how force induces PDL progenitor cell pyroptosis, thereby influencing OTM and alveolar bone remodeling is unclear. In this study, we revealed a novel mechanism that mechanical force induced pyroptosis in periodontal tissues and PDL progenitor cells, which further promoted OTM and alveolar bone remodeling. The functional role of the force-induced pyroptosis depended on Caspase-1 and activated the TRPV4 signaling.

The role of pyroptosis has been primarily studied in phagocytes, which is initiated by inflammatory caspases and leads to GSDMD-induced pore formation and cleavage of pro-inflammatory cytokine IL-1β.^19^ Recently, pyroptosis was also observed in the inflammatory-related diseases including arthritis, myocarditis and bacterial-induced periodontitis.^20–22^ OTM is an aseptic inflammatory reaction and alveolar bone remodeling process activated by mechanical stimuli, characterizing by bone resorption in the compression side and bone apposition in the tension side.^6,9^ We previously found that during OTM, various inflammatory cytokines, chemokines, and the activations of immune cells were detected.^7,8^ However, the underlying mechanism has not been explored. In this study, we revealed that mechanical force could induce Caspase-1-dependent pyroptosis in PDL progenitor cells, which contributes to OTM and alveolar bone remodeling. Previous studies have found that cyclic stretch could induce pyroptosis in PDL cells.^14,23^ Consistent with the previous findings, the present study shows a novel finding that mechanical force could induce pyroptosis in PDL progenitor cells, which contributes to OTM and alveolar bone remodeling. Nevertheless, the activation of osteoclastic activity by pyroptosis may influence root resorption, which needs further investigation in future studies.

Depending on different environmental stimuli, pyroptosis can be divided into canonical and non-canonical types. In canonical pyroptosis, NLRP3 inflammasomes bind to Caspase-1 and activate Cleaved-Casp-1 to cleave GSDMD and execute pyroptosis via pore-forming activity.^2^ In non-canonical pyroptosis, Caspase-11/4/5 was activated to cleave GSDMD upon recognition of cytosolic lipopolysaccharide (LPS), which is independent of inflammasomes and Caspase-1.^24^ Force-induced OTM was an aseptic inflammatory reaction, which was different from LPS-induced inflammatory process.^9^ In this study, we confirmed that force-induced pyroptosis required the activation of Caspase-1. *Caspase-1*^−/−^ mice showed reduced expressions of pyroptosis markers and decreased number of TRAP^+^ osteoclasts compared with WT mice. Consistently, blocking the Caspase-1 level by the application of Caspase-1 inhibitor VX765 also decreased the expressions of pyroptosis-related markers in PDL progenitor cells and the ratio of RANKL/OPG compared with the force group. These results suggest that Caspase-1-dependent pyroptosis contribute to force-induced OTM and alveolar bone remodeling.

So far, how mechanical force induced pyroptosis remains unclear. TRPV4, a typical mechanosensitive channel, is associated with force-induced alveolar bone remodeling processes.^16,17^ Our previous study found that TRPV4 was activated in force-induced PDL progenitor cells, which contributed to the modulation of PDL progenitor cells function and regulated alveolar bone remodeling.^17^ Interestingly, TRPV4 was recently reported to be involved in some pyroptosis-related diseases.^18^ In this study, we showed that TRPV4 activation under mechanical force contributed to the induction of Caspase-1-dependent canonical pyroptosis in PDL progenitor cells. Inhibiting TRPV4 could suppress the expressions of pyroptosis-related markers, decrease force-induced Ca^2+^ influx, suppresse ROS expression, and reverse the repression of mitochondrial membrane potential and mitochondrial damage induced by force.

The phenomenon that the pyroptosis genes remain upregulated in ex vivo PDL progenitor cells is very interesting. Previous studies have found that external stimulus including stress, nutrients and pathogens could trigger transcriptional memory in many cells, which was defined as a phenomenon that transient gene activation by a variety of external signals results in a heritable primed state that is maintained in the absence of active transcription.^25^ Our previous study has also demonstrated that mechanical force in vivo could change the characteristics of rat primary PDL progenitor cells including promoting their proliferation, pro-inflammatory cytokine expression and immunoregulation.^17^ In this study, increased expressions of pyroptosis related markers were detected in ex-vivo human PDL progenitor cells with force stimuli, which is consistent with the previous findings. The mechanism of how the PDL progenitor cells possess stimulus memory needs our further exploration.

**Fig. 8:**
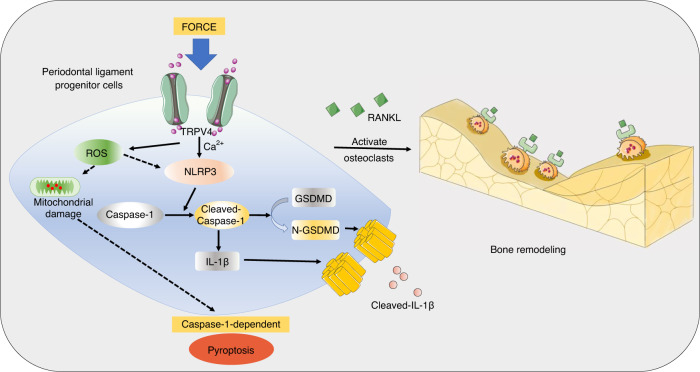
Schematic showing force-induced Caspase-1-dependent pyroptosis in PDL progenitor cells via TRPV4 signaling, ultimately contributing to the activation of osteoclastogenesis and alveolar bone remodeling

In summary, these data indicate that mechanical force induces Caspase-1-dependent pyroptosis in PDL progenitor cells in rat, mice and human models. This Caspase-1-dependent pyroptosis contributes to OTM and alveolar bone remodeling (Fig. 8). This study provides a novel insight into the modulation of osteoclastogenesis under mechanical stimuli. It suggests that targeting Caspase-1-dependent pyroptosis may be a promising strategy to accelerate OTM.

## Materials and methods

### Animals and orthodontic force treatment

6–8-week-old Male Sprague Dawley rats (body weight of 200–250 g) and C57BL/6N mice were obtained from Weitong Lihua Experimental Animal Center (China), and *Caspase-1*^*−/−*^ mice were generated by Viewsolid Biotech (Beijing, China). They were housed in controlled animal facilities with a temperature of (23 ± 2) °C, a humidity of 40% to 65%, and a 12/12 h light/dark cycle. Animals were fed with a standard laboratory diet and allowed ad libitum access to drinking water. All animals were maintained in specific pathogen-free (SPF) cages randomly and fed a normal diet. The animal number in each group (*n* = 3–6) is estimated according to our pre-experiment.^12^ Humane care was provided to each animal during the experiments according to the criteria outlined in the Guide for the Care and Use of Laboratory Animals published by the National Institutes of Health. Six- to eight-week-old male Sprague-Dawley rats, C57BL/6 N mice, and *Caspase-1*^*−/−*^ mice were used in the study. All the protocols were approved by the Peking University Ethical Committee (LA2013-92). The study conforms to the ARRIVE guidelines.

Mechanical force was applied to rats or mice as previously described.^26^ Briefly, in rats, nickel-titanium coil springs of 0.2 mm in wire size, 1 mm in diameter, and 4 mm in length (Smart Technology) were ligated between the maxillary right first molar and the maxillary incisors to provide 50–60 g force for 3 d, 7 d and 14 d.^7,27^ The maxillary left first molar without force application served as the control. Five rats were used at each time point. Another 3 rats received force application for 7 d, and the periodontal tissues including the alveolar bone and periodontal ligament of the mesial side of the maxillary first molars were collected for the detection of gene expressions.

In mice, nickel-titanium coil springs with the same size and 1 mm in length were ligated in a similar way to provide 20–30 g force for 7 d.^12,28,29^ The contralateral first molar served as control. Mice were randomly divided into four groups as follows: (i) Force: force loading and 0.9% NaCl treatment every two days; (ii) Force + PPVI: force loading and pyroptosis activator Polyphyllin VI (PPVI, S9302, Selleck) treatment (5 mg/kg every two days); (iii) Force + MCC950: force loading and pyroptosis inhibitor MCC950 (S7809, Selleck) treatment (20 mg/kg every two days); (iv) Control: the group without force loading and treatment. Drugs were injected intraperitoneally (i.p.).^30,31^ Each group comprised 5 mice. In addition, force was also applied to the *Caspase-1*^*−/−*^ mice for 7d to compare the difference of OTM and alveolar bone remodeling with the wild-type mice (*n* = 5).

After OTM, the animals were sacrificed and the maxillae were harvested for micro-CT scanning and histological staining. For histological staining, consecutive horizontal sections (4 μm) were obtained from the middle to apical third of each maxillary first molar. The sections from similar position of the roots were used for histological study.

### Micro-CT scanning and measurement of orthodontic tooth movement (OTM) distance

The animals were sacrificed by overdose of pentobarbital sodium, and the maxillae were harvested, fixed in 4% paraformaldehyde (PFA), and scanned by micro-CT system (Inveon MMCT, Berlin, Germany) at 80 kV, 500 µA, and an image voxel size of 18 µm. Mimics 13.1 software (Materialise, Leuven, Belgium) was used for 3D image reconstruction and segmentation. The distance of OTM was measured from the occlusal view of the maxillae in 3D images using a modified method described previously.^7^ Briefly, the distance between the midpoint of the first molar distal-marginal ridge and the midpoint of the second molar mesial-marginal ridge was measured by a trained researcher who was blinded to the group assignment. The average of the three measurements was calculated as the tooth movement distance.

### Tartrate-resistant acid phosphatase (TRAP) staining

TRAP staining was utilized to detect the number of osteoclasts using an acid phosphatase kit (387A-1KT; Sigma) according to the manufacturer’s protocol. The number of TRAP-positive multinucleated (>3 nuclei) osteoclasts in five visual fields at 20× magnification in each well was counted. The final results came from the average of 5 independent tests. In histological sections, the number of TRAP-positive multinucleated (>3 nuclei) osteoclasts in five visual fields at 40× magnification in each histological section was counted. The final results came from the average of 5 independent tests.

### Immunohistochemical staining, immunofluorescence staining

For immunohistochemical staining, after mice sacrifice, the trimmed maxillae were fixed in 4% PFA for 24 h. After decalcifying in ethylenediaminetetraacetic acid for 4 weeks, the tissues were embedded in paraffin. 4-μm consecutive horizontal sections were obtained from the middle to apical third of the roots, and sections from the similar positions were chosen. Immunohistochemistry was performed with a two-step detection kit (Zhongshan Golden Bridge Biotechnology, Beijing, China) as previously described.^7^ Primary antibodies included anti-GSDMD (1:200; AF4012, Affinity), anti-Caspase-1 (1:200; AF5418, Affinity), and anti-IL-1β (1:200; AF5103, Affinity). Histological changes in stained tissues were observed using an optical microscope (Olympus, Japan). The positive staining cells were counted in five different slides from each sample.

Immunofluorescence staining was performed as previously described.^32^ The sections were incubated with primary antibodies including anti-CD90 (1:200; ab225, Abcam), anti-GSDMD (1:200; AF4012, Affinity), anti-Caspase-1 (1:200; AF5418, Affinity), anti-IL-1β (1:200; AF5103, Affinity) to observe the numbers of Caspase-1^+^CD90^+^ cells, GSDMD^+^CD90^+^ cells, and IL-1β^+^CD90^+^ cells in the compression side of the periodontal tissues after force loading; antibodies including anti-TRPV4 (1:200; ab39260, Abcam), anti-GSDMD (1:200; SC-393581, Santa Cruz), anti-Caspase-1 (1:200; SC-392736, Santa Cruz) were used to observe the numbers of Caspase-1^+^TRPV4^+^ cells and GSDMD^+^TRPV4^+^ cells in the compression side of the periodontal tissues after force loading. Then, sections were incubated with fluorescein isothiocyanate-conjugated or tetramethylrhodamine isothiocyanate-conjugated secondary antibodies (1:200; Zhongshan Golden Bridge Biotechnology, Beijing, China). Nuclei were counterstained with 4′,6-diamidino-2-phenylindole (DAPI, P0131, Beyotime). Confocal images were processed with LSM 5 Release 4.2 software after acquisition by a laser-scanning microscope (LSM510; Zeiss, Germany). The cells double-stained by red and green immunofluorescence were calculated. The positively double-stained cells were counted in five different slides from each sample. The final results came from the average of 5 independent samples.

### Human PDL (h-PDL) progenitor cell isolation ex vivo

The volunteers planning to extract four premolars due to orthodontic treatment demands were included. The h-PDL progenitor cells were isolated from the upper premolars of receiving orthodontic force for 7 d (hF7d) representing active force stimulus.^33^ The h-PDL progenitor cells from the lower premolars without force loading from the same patient served as controls. Six teeth of three patients were isolated in each group (*n* = 3). The clinical procedures were approved by Peking University Ethical Committee (PKUSSIRB-201311103) and informed consent was signed by the patients. Briefly, the periodontal ligament scraped from the root surface of the premolars with or without force stimuli were digested in a mixture of 3 mg/mL type I collagenase (Worthington Biochem, USA) and 4 mg/mL dispase II (Roche, Germany) for 1 h at 37 °C. The single cell suspensions were obtained and used for cell culture with a-MEM medium (Invitrogen, USA) with 20% fetal bovine serum (Gibco, USA) and 1% Penicillin/Streptomycin (Gibco, USA). When the single cell suspensions adhered to the wall for 3 days, the primary cells were digested and cultivated on the six-well plate for further experiments.

### Mechanical loading and treatments on human PDL progenitor cells in vitro

Human PDL progenitor cells were isolated as previously described and were identified following previous protocols,^34^ which used at passage 4. Compressive force loading was provided by glass layers and 50 mL plastic tube caps containing weighed metal balls as previously described.^35,36^ 1.5 g/cm^2^ compressive force was applied to PDL progenitor cells for different time points (3–24 h), and different compressive force (0.5–2.0 g/cm^2^) was applied to PDL progenitor cells for 6 h. In addition, after being subjected to 1.0 g/cm^2^ and 1.5 g/cm^2^ compressive force for 6 h, PDL progenitor cells were collected for further experiments of optical microscope (OM, Olympus, Japan), scanning electron microscope (SEM) and transmission electron microscope (TEM).

To confirm the influence of pyroptosis under mechanical stimuli, pyroptosis activator PPVI (4 μmol/L), pyroptosis inhibitor MCC950 (10 μmol/L) and Caspase-1 inhibitor Belnacasan (VX765, 20 μM, S2228, Selleck) were added to PDL progenitor cells for 18 h in advance, then 1.5 g/cm^2^ force was applied to PDL progenitor cells for 6 h.^31,37^ In addition, TRPV4 inhibitor GSK219 (10 mmol/L, Selleck) were applied to PDL progenitor cells for 1 h and then stimulated with force loading (1.5 g/cm^2^, 6 h).^17^ PDL progenitor cells without force-loaded and drug treatment served as controls.

### Co-culture of PBMCs and PDL progenitor cells

H-PBMCs were selected from peripheral blood. The h-PDL progenitor cells of passage 1 (5 × 10^3^ cells per mL) with or without orthodontic force stimuli were seeded into 24-well plates to co-cultured with h-PBMCs (1 × 10^6^ cells per mL). Macrophage colony-stimulating factor (MCS-F, 30 ng/mL) and soluble receptor activator of nuclear factor–κB ligand (sRANKL, 50 ng/mL) were added to the cultured medium. After co-culturing for 14 days, cells were fixed and stained with an acid phosphatase kit (387A-1KT; Sigma) for tartrate-resistant acid phosphatase (TRAP) staining.

### Immunocytofluorescense staining

Immunocytofluorescense staining was performed according to a previously described method.^11^ Briefly, cells were fixed in 4% paraformaldehyde and permeabilized with 0.1% Triton X-100 at room temperature for 10 min. Next, the cells were incubated with 5% Bovine Serum Albumin (BSA) at room temperature for 1 h, followed by incubation with anti-CD90 (1:200; SC-53456, Santa Cruz), anti-TRPV4 (1:200; ab39260, Abcam), anti-GSDMD (1:200; AF4012, Affinity), anti-Caspase-1 (1:200; AF5418, Affinity), and anti-IL-1β (1:200; AF5103, Affinity) at 4 °C overnight. After thoroughly washed, the cells were then incubated with fluorescein isothiocyanate-conjugated or tetramethylrhodamine isothiocyanate-conjugated secondary antibodies (1:200; Zhongshan Golden Bridge Biotechnology, Beijing, China) in the dark at room temperature for 1 h. Nuclei were counterstained with DAPI (P0131, Beyotime, China). Confocal microscopic images were processed with LSM 5 Release 4.2 software after acquisition by a laser-scanning microscope (LSM510; Zeiss, Germany). The positively stained cells were counted in five different slides from each sample.

### Quantitative real-time polymerase chain reaction (PCR)

The rat periodontal tissues included the alveolar bone and periodontal ligament were separated from the mesial side of first molars. Tissues were collected in 1.5 mL EP tube with 1 mL TRizol reagent (Invitrogen, Carlsbad, CA), and smashed for 5 min. Then tissues were centrifuged and the supernatant was collected. For PDL pregenitors in vitro, they were washed by PBS and added TRizol reagent. Total RNA was extracted from cultured cells or periodontal tissues with TRizol reagent (Invitrogen, Carlsbad, CA) following the manufacturer’s protocol. 2 μg of RNA was reverse transcribed into complementary first-strand cDNA using cDNA synthesis kits (Takara Bio, Inc., Otsu, Japan). Then real-time Polymerase Chain Reaction (PCR) was performed using the FastStart Universal SYBR Green master kit (Roche) on an Applied Biosystems 7500 real-time PCR System (Life Technologies Corporation, United States) to determine the relative mRNA expression level. Fold changes of target genes were calculated with ΔCT method using GAPDH or β-actin as a reference control. The sequences of primers were designed by Primer Premier 5.0 software and were listed as follows:

Human:

*GAPDH* sence/antisence: 5′- TGCCACTCAGAAGACTGTGG-3′/5′- TTCAGCTCTGGGATGACCTT-3′.

*NLRP3* sence/antisence:5′-CCACAAGATCGTGAGAAAACCC-3′/5′- CGGTCCTATGTGCTCGTCA-3′

*Caspase-1* sence/antisence:5′- CGTTCCATGGGTGAAGGTACA-3′/5′- TGCCCCTTTCGGAATAACGG-3′

*GSDMD* sence/antisence:5′-GTGTGTCAACCTGTCTATCAAGG-3′/5′- CATGGCATCGTAGAAGTGGAAG-3′

*IL-1β* sence/antisence:5′- TTCGACACATGGGATAACGAGG-3′/5′- TTTTTGCTGTGAGTCCCGGAG-3′

*RANKL* sence/antisence:5′- ATCAGAGCAGAGAAAGCGATG-3′/5′-GACTCACTTTATGGGAACCAG-3′

*OPG* sence/antisence:5′- TTGAAATGGCAGTTGATTCCTTT -3′/5′- TATCCTCTTTCTCAGGGTGCTTG-3′

*CTSK* sence/antisence:5′-ATCCGGACTGTGACGAGTTG -3′/5′-ATTTGGGAGCAGCTGGGATG-3′

*TRAP* sence/antisence: 5′-ACTACCAGAAACGAGTGGGAA-3′/5′-GCATCTGTTCTCGGAAAACCT-3′

Rat:

*β-actin* sence/antisence:5′- TGACAGGATGCAGAAGGAGA-3′/5′- TAGAGCCACCAATCCACACA-3′

*Nlrp3* sence/antisence:5′- TCACGTCTTGAAGCCACATCC-3′/5′- GAAGCAAAGTTCCTCCAGACAG-3

*Caspase-1*:5′- GTGGTTCCCTCAAGTTTTGC-3′/5′-CCGACTCTCCGAGAAAGATG-3′

*Gsdmd* sence/antisence:5′- CCAACATCTCAGGGCCCCAT-3′/5′-TGGCAAGTTTCTGCCCTGGA-3′

*Il-1β* sence/antisence:5′- CACCTCTCAAGCAGAGCACAG-3′/5′- GGGTTCCATGGTGAAGTCAAC-3′

*Trpv1* sence/antisence:5′-GCCGCTGAACCGACTC-3′/5′-CCCATCTGCTGGAAAC-3′

*Trpv2* sence/antisence:5′- CGCCATTGAGAAGAGGAGTC-3′/5′- GCTTACCACATCCCACTGCT-3′

*Trpv3* sence/antisence:5′- GCGTGGAGGAGTTGGTAGAG-3′/5′- CTCTGTGTACTCGGCGTTGA-3′

*Trpv4* sence/antisence:5′- CAGGTGGGGAGGCTTTT-3′/5′- GCGGCTGCTTCTCTATG-3′

### Western blotting

Western blottings were performed as previously described.^17^ Cells were lysed with RIPA buffer (Thermo Fisher Scientific) and the total proteins were harvested. A Pierce BCA protein assay kit (Thermo Fisher Scientific) was used to perform the protein quantification. Total protein (30 μg) was separated by 10% SDS–polyacrylamide gel and then transferred onto a polyvinylidene difluoride (PVDF) membrane (Millipore). After being blocked in 5% BSA for 1 h at room temperature, the membranes were incubated overnight at 4 °C with primary antibodies including GAPDH (1:5 000, AF7021, Affinity), NLRP3 (1:1 000, PA5-79740, Thermo Fisher), Caspase-1 (1:500, AF5418, Affinity), Cl-Casp-1 (1:300, AF4005, Affinity), GSDMD and N-GSDMD (1:500, AF4012, Affinity), IL-1β (1:500, AF5103, Affinity), Cl-IL-1β (1:300, AF4006, Affinity), RANKL (1:500, AF0313, Affinity), OPG (1:500, DF6824, Affinity), TRPV4 (1:1 000, ab39260, Abcam). The blots were then incubated with a horseradish peroxidase-conjugated secondary antibody (1:5 000; Zhongshan Golden Bridge Biotechnology, Beijing, China). The membranes were washed three times with 0.1% TBS Tween (P9416, Sigma-Aldrich). The bands were detected using enhanced chemiluminescence (34577, Thermo Fisher Scientific), and BioMax film (Kodak, Rochester, New York, USA) was used to detect the immunoreactive proteins. The relative density of at least three independent results was measured by Image J software. All the western blotting results were the average of 3 independent experiments.

### Enzyme-linked immunosorbent assay (ELISA)

RANKL and IL-1β concentrations in culture supernatants were detected by ELISA (mlbio, China) following the manufacturer’s instructions. The results were determined by comparing the samples to the standard curve generated by the kit. All samples and standards were assayed in triplicate.

### Scanning electron microscopy (SEM) and transmission electron microscopy (TEM)

For SEM, the PDL pregenitor samples were pre-fixed in 2.5% glutaraldehyde in PBS (pH 7.4) at 4 °C for 12 h and washed with PBS three times. The samples were dehydrated in a graded series of ethanol solutions and critical-point dried, and then sputter-coated with gold for 2 min at 20 mA. The PDL pregenitor samples were observed using SEM (Hitachi S-4800, Japan) at 10 kV.

For TEM, PDL pregenitors were harvested, washed three times with PBS, and fixed in 2.5% glutaraldehyde for 2 days at 4 °C. PDL pregenitors were post-fixed in 1% osmium tetroxide for 2 h. After they were dehydrated using a graded series of ethanol solutions, the samples were embedded in Embed-812 resin and cut into ultrathin sections (70 nm thick). The ultrathin sections were installed on a copper wire and stained with dioxyuranium acetate and lead citrate. These sections were examined with TEM (JEM-100CX, Japan) at 100 kV.

### Ca^2+^ influx measurement

The calcium-regulated fluorescent intracellular calcium indicator, Fluo-4 acetoxymethyl ester form (Fluo-4 AM, F8500, Solarbio, China) was used to monitor real-time elevations of intracellular calcium following force stimuli and the inhibition of TRPV4, according to the manufacturer’s instructions. Briefly, PDL pregenitors of different groups (Control, Force, Force+GSK219) were loaded with 4 × 10^−4^ mol/L Fluo-4 AM fluorescent dye diluted 1/200 in Ca^2+^ free Hank’s buffered salt solution (HBSS) for 60 min at room temperature. After this period, cells were washed two times with HBSS and further incubated with 300 µL of HBSS for 60 min. Cells were stained with Hoechst 33342 (C1027, Beyotime, China) in the dark for 20 min. Then, Fluo-4 AM positive cells were photographed by confocal microscopy (LSM510; Zeiss, Germany), and the images were processed using LSM 5 Release 4.2 software.

### Measurement of intracellular reactive oxygen species (ROS)

The content of intracellular ROS was detected by the H2DCF-DA fluorescence probe (Beyotime, China) according to the manufacturer’s instructions. After that PDL pregenitors of different groups (Control, Force, Force+GSK219) were incubated with 10 mM DCFH-DA for 20 min at 37 °C in the dark, PDL pregenitors were washed twice with serum-free medium and resuspended with a-MEM medium including Hoechst 33342 (C1027, Beyotime, China). The intracellular ROS was assessed at 488/525 nm using fluorescent microscopy (Leica, Germany) and analyzed by Image-Pro Plus 6.0 software (Media Cybernetics, MD, USA).

### Mitochondrial morphology detection and mitochondrial membrane potential (Δψm)

Mitochondrial morphology was detected by Mito-tracker dyes. Mitochondria were labeled with the MitoTracker Red (C1049B, Beyotime, China) for 30 min in the dark. The mitochondrial morphology was photographed by a confocal microscope (LSM510; Zeiss, Germany).

The Δψm was analyzed using the fluorescent probe JC-1 assay kit (C2003S, Beyotime, China) according to the manufacturer’s instructions. JC-1 exhibits red fluorescence aggregates in the mitochondrial matrix in normal cells. When the Δψm is reduced, monomeric JC-1 displays green fluorescence. Therefore, the rate of green/red fluorescence was used to represent the Δψm in each cell sample. PDL pregenitors of different groups (Control, Force, and Force + GSK219) were cultured on the coverslips in 12-well plates and loaded with JC-1 (1:400 dilution) at 37 °C for 20 min. The images were observed and captured under a fluorescence microscope (Leica, Germany).

### Adenosine triphosphate (ATP) Assay

ATP levels were measured using the ATP bioluminescence detection kit (S0026, Beyotime, China). Briefly, PDL pregenitors were lysed with a lysis buffer supplied with the kit and centrifuged at 12 000 × *g* for 5 min at 4 °C. The supernatant was collected for ATP detection. The protein concentration of the supernatant was measured using the BCA Protein Assay Kit (P0012S, Beyotime, China). Furthermore, 100 µL supernatant with 100 µL ATP detection buffer was measured using a microplate reader (EnSpire, USA). The standard solution was diluted in gradient to obtain the standard curve (1 nmol/L-1 µmol/L). Then, ATP levels were calculated according to standard curves and normalized according to standard protein concentrations.

### Statistical analysis

Statistical analysis was performed with GraphPad Prism 9.0 software. Data were presented as mean ± standard deviation (SD). Statistical differences between two groups were assessed by a two-tailed independent Student’s *t* test, and statistical differences among three and more groups were assessed by one-way analysis of variance (ANOVA). Tukey’s multiple-comparison test was used for the post hoc comparison of ANOVA. A *p*-value less than 0.05 represented statistically significant.

### Supplementary information

Supporting Information

## Data Availability

All data associated with this study are presented in the paper.
