# Supplementary material for: Force-induced Caspase-1-dependent pyroptosis regulates orthodontic tooth movement
Source: Int J Oral Sci. 2024 Jan 15;16:3. doi: 10.1038/s41368-023-00268-7 (PMC10788340; doi:10.1038/s41368-023-00268-7)
Supplement: Supplementary file 1 — Supporting Information [file 41368_2023_268_MOESM1_ESM.docx]

**Supporting Information**

**Force-induced Caspase-1-dependent pyroptosis regulates orthodontic tooth movement**

**Running title：Pyroptosis regulates tooth movement**

Liyuan Chen^1^, Huajie Yu^2^, Zixin Li^1^, Yu Wang^1^, Shanshan Jin^1^, Min Yu^1^, Lisha Zhu^1^, Chengye Ding^1^, Xiaolan Wu^1^, Tianhao Wu^1^, Chunlei Xun^1^, Yanheng Zhou^1^, Danqing He^1*^, Yan Liu^1*^

^1^Department of Orthodontics, Central Laboratory, Peking University School and Hospital for Stomatology & National Center for Stomatology & National Clinical Research Center for Oral Diseases & National Engineering Research Center of Oral Biomaterials and Digital Medical Devices & Beijing Key Laboratory of Digital Stomatology & Research Center of Engineering and Technology for Computerized Dentistry Ministry of Health & NMPA Key Laboratory for Dental Materials & National Engineering Research Center of Oral Biomaterials and Digital Medical Devices, Beijing 100081, China

^2^Peking University Hospital of Stomatology Fourth Division

^*^Corresponding author. Email: [hedanqing@bjmu.edu.cn](mailto:hedanqing@bjmu.edu.cn) (D.H.), orthoyan@bjmu.edu.cn (Y.L.)


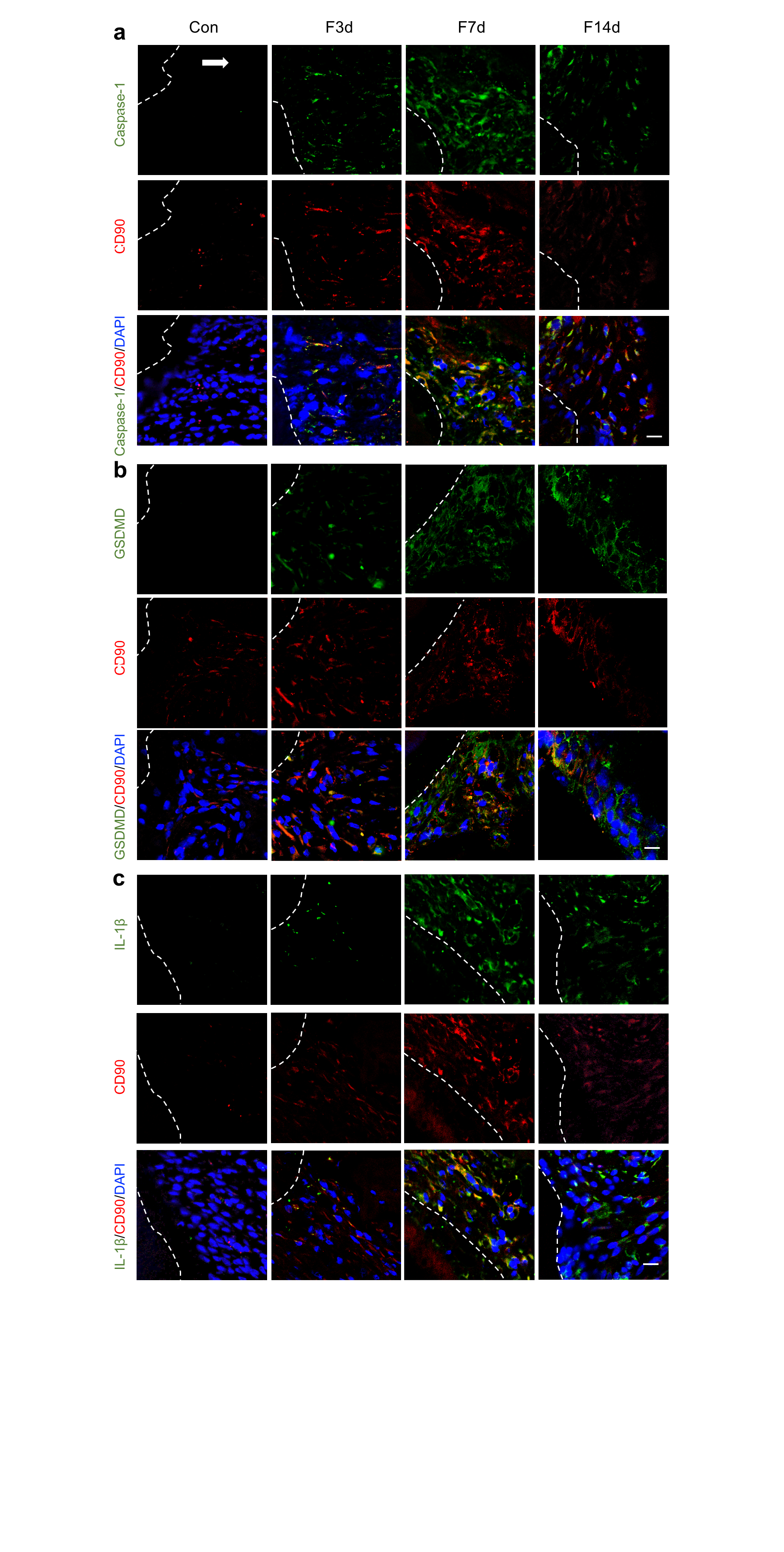


**Figure S1. Representative immunofluorescence images of Fig. 1b.** The number of Caspase-1-positive (green) and CD90-positive (red) double-stained cells (merged yellow), GSDMD-positive (green) and CD90-positive (red) double-stained cells (merged yellow) and Il-1β-positive (green) and CD90-positive (red) double-stained cells (merged yellow) increased after force loading for 3 d, 7 d and 14 d. Dashed lines mark the outline of distobuccal roots. Arrow represents the direction of the force. Scale bar: 50 μm.

^
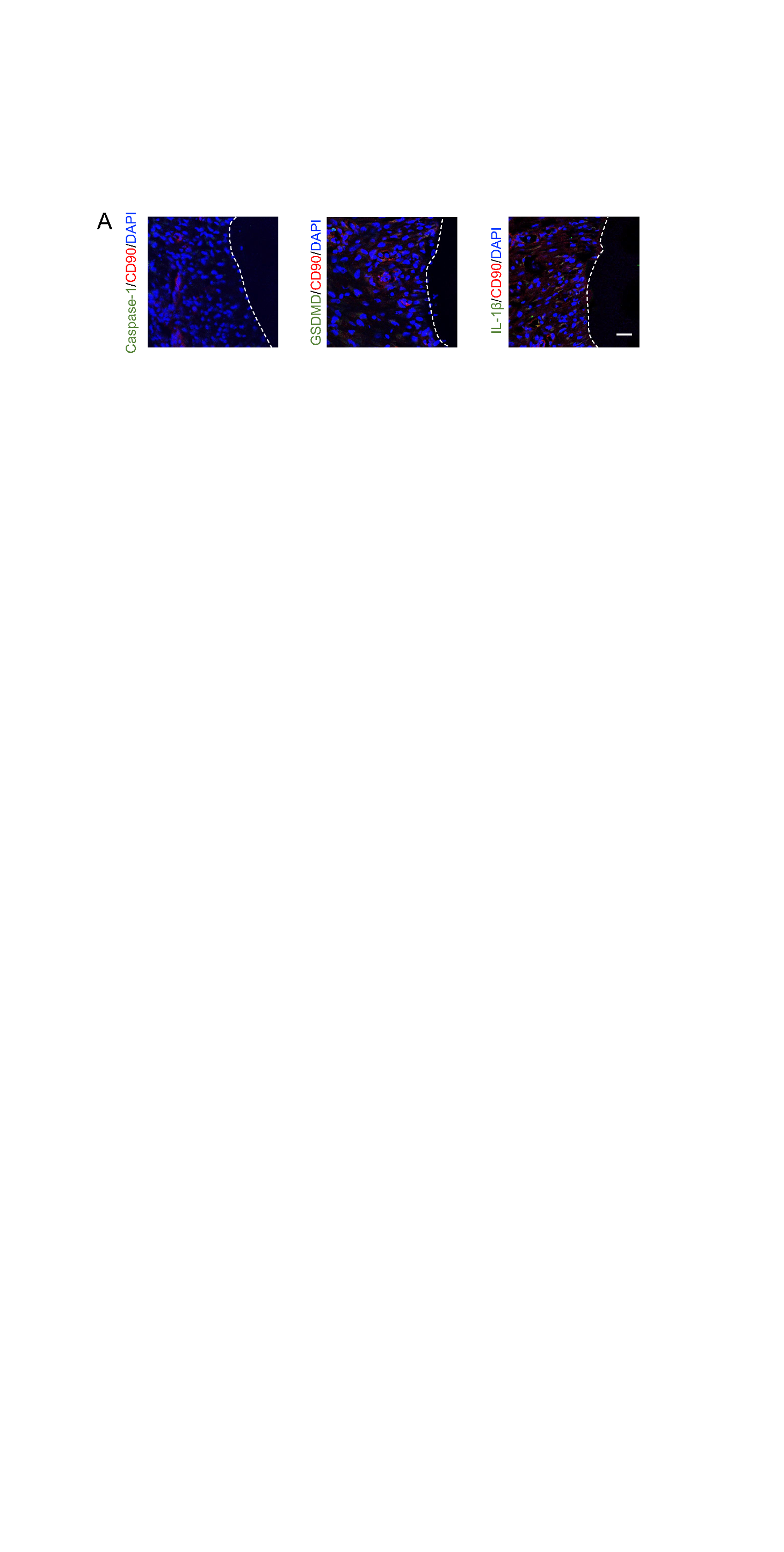
^

**Figure S2. Representative immunofluorescence images on the tension side of distobuccal roots in rats with force loading for 7d.** No obvious Caspase-1-positive (green) and CD90-positive (red) double-stained cells (merged yellow), GSDMD-positive (green) and CD90-positive (red) double-stained cells (merged yellow) and Il-1β-positive (green) and CD90-positive (red) double-stained cells (merged yellow) were detected. Dashed lines mark the outline of distobuccal roots. Scale bar: 50 µm.


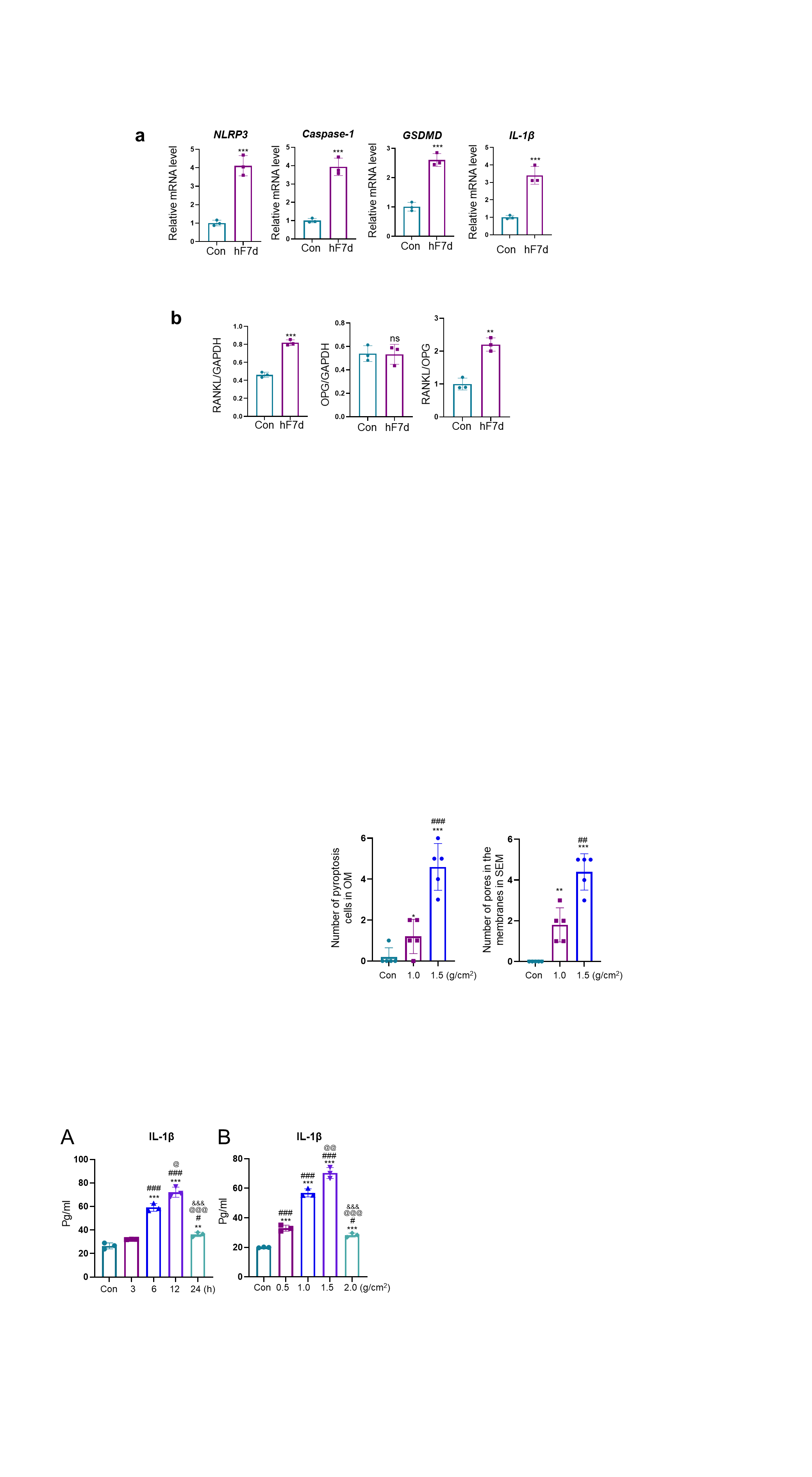


**Figure S3. Pyroptosis is activated in *ex-vivo* force-stimulated human PDL progenitor cells and regulates osteoclastic activity**. **a** Real time-PCR of *Nlrp3, Caspase-1, Gsdmd,* and *Il-1β* in in *ex-vivo* h-PDL progenitor cells receiving force stimulation for 7 d. *n* = 3 biologically independent samples. **b** Semiquantification analysis of western blotting results of RANKL and OPG expressions in *ex-vivo* h-PDL progenitor cells. ***P* < 0.01, ****P* < 0.001, ns > 0.05 versus Con. *n* = 3 biologically independent samples.


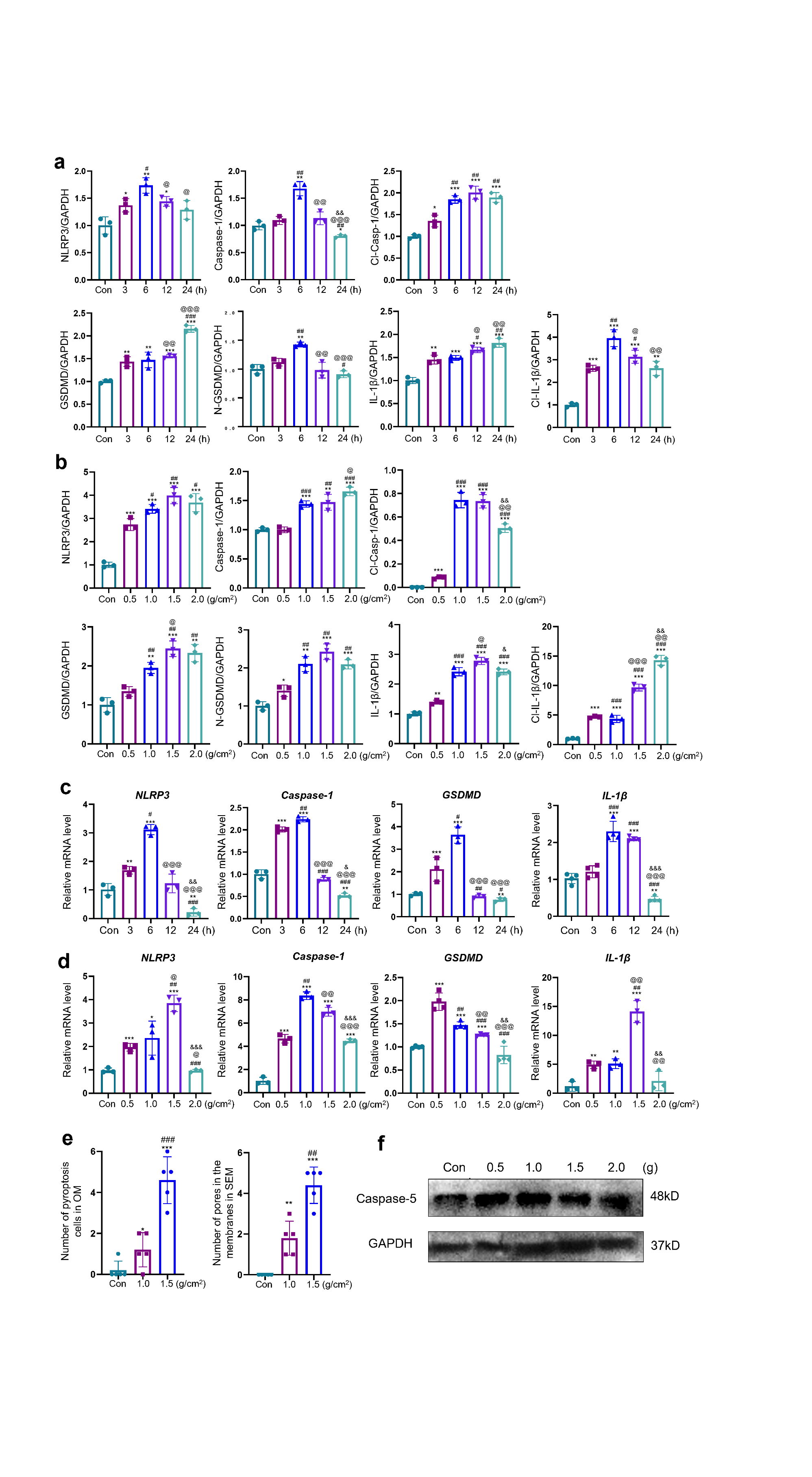


**Figure S4. Force-induced pyroptosis in human PDL progenitor cells *in vitro***. **a** Semi-quantification results of Fig. 4**h** (western blotting of pyroptosis-related proteins in PDL pregenitors under 1.5g /cm^2^ mechanical force at different time points). **P* < 0.05, ***P* < 0.01, ****P* < 0.001 versus Con; #*P* < 0.05, ##*P* < 0.01, ###*P* < 0.001 versus 3 h; @*P* < 0.05, @@*P* < 0.01, @@@*P* < 0.001 versus 6 h; &&*P* < 0.01 versus 12 h. **b** Semi-quantification results of Fig. 4**i** (western blotting of pyroptosis-related proteins in PDL pregenitors under different force for 6 h). ***P* < 0.01, ****P* < 0.001 versus Con; #*P* < 0.05, ##*P* < 0.01, ###*P* < 0.001 versus 0.5 g/cm^2^; @*P* < 0.05, @@*P* < 0.01, @@@*P* < 0.001 versus 1.0 g/cm^2^; &*P* < 0.05, &&*P* < 0.01 versus 1.5 g/cm^2^. **c** Real time-PCR of *NLRP3, GSDMD, Caspase-1* and *IL-1β* in PDL progenitor cells under 1.5 g/cm^2^ compressive force at different time points. ***P* < 0.01, ****P* < 0.001 versus Con; #*P* < 0.05, ##*P* < 0.01, ###*P* < 0.001 versus 3h; @@@*P* < 0.001 versus 6 h; &*P* < 0.05, &&*P* < 0.01, &&&*P* < 0.001 versus 12 h. **d** Real time-PCR of *NLRP3, GSDMD, Caspase-1* and *IL-1β* under different force values for 6 h. **P* < 0.05, ***P* < 0.01, ****P* < 0.001 versus Con; ##*P* < 0.01, ###*P* < 0.001 versus 0.5 g/cm^2^; @*P* < 0.05, @@*P* < 0.01, @@@*P* < 0.001 versus 1.0 g/cm^2^; &&*P* < 0.01, &&&*P* < 0.001 versus 1.5 g/cm^2^. **e** Semi-quantification results of Fig. 4**j** (the number of pyroptosis cells in OM and the number of pores in the membranes in SEM under 1.0 g/cm^2^ and 1.5 g/cm^2^ force for 6 h). ***P* < 0.05, ***P* < 0.01, ****P* < 0.001 versus Con; ##*P* < 0.01, ###*P* < 0.001 versus 1.0 g/cm^2^. **f** Western blotting of Caspase-5 of non-canonical pyroptosis in PDL pregenitors under different force values for 6 h. Results were presented as mean ± SD. n = 3 independent experiments.


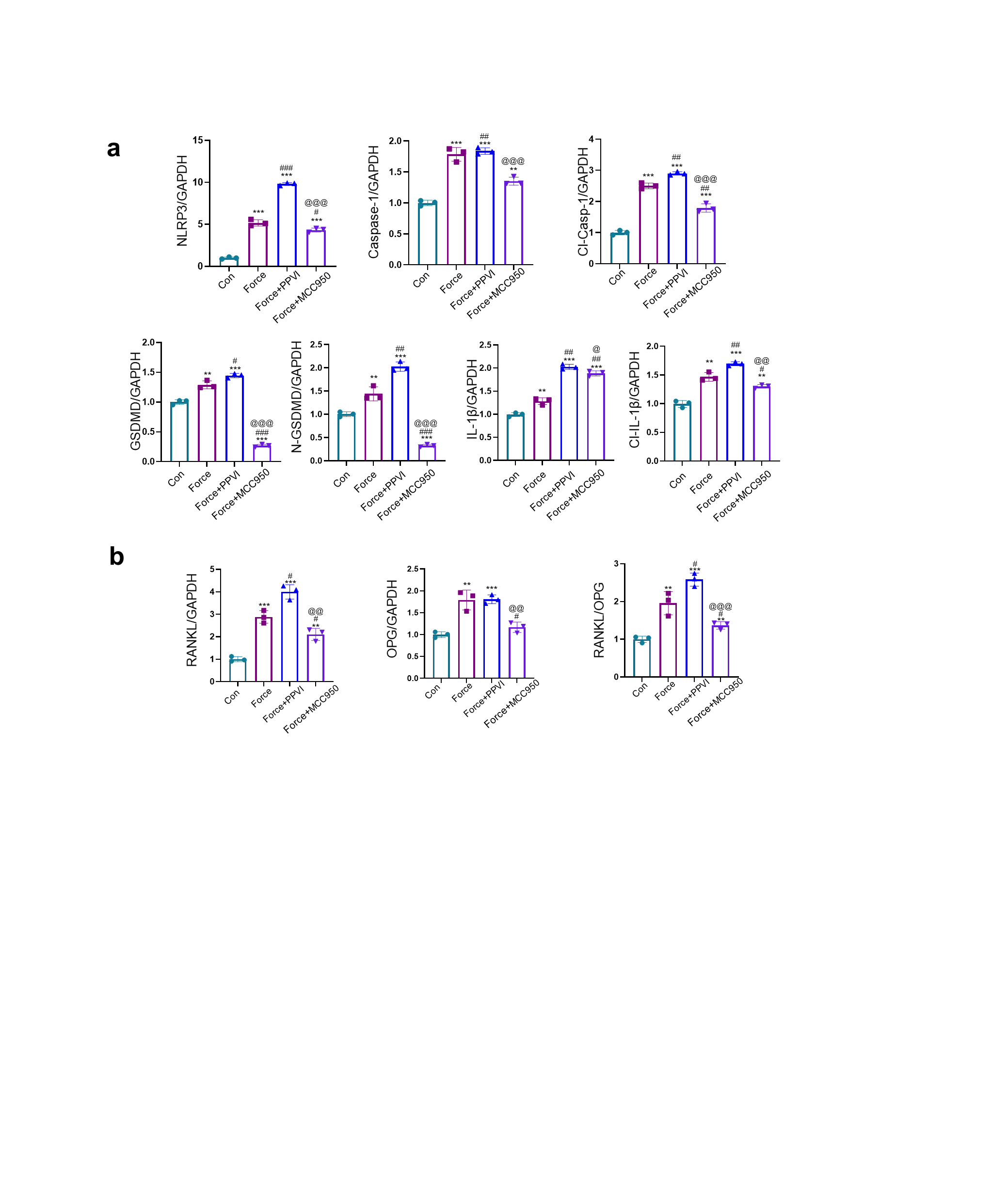


**Figure S5. Regulation of PDL progenitor cell pyroptosis influences osteoclastic activity *in vitro*.** **a** Semiquantification analysis of Fig. 5**a** in PDL progenitor cells under 1.5 g/cm^2^ force loading for 6 h with or without the appliance of PPVI and MCC950 *in vitro*. GAPDH served as an internal control for equal loading. **b** Semiquantification analysis of Fig. 5**c** (western blotting of RANKL and OPG in PDL progenitor cells under 1.5 g/cm^2^ mechanical force for 6 h with or without the appliance of PPVI and MCC950). **P* < 0.05, ***P* < 0.01, ****P* < 0.001 versus Con; #*P* < 0.05, ##*P* < 0.01, ###*P* < 0.001 versus Force; @@*P* < 0.01, @@@*P* < 0.001 versus Force+PPVI. Results were presented as mean ± SD. *n* = 3 independent experiments.


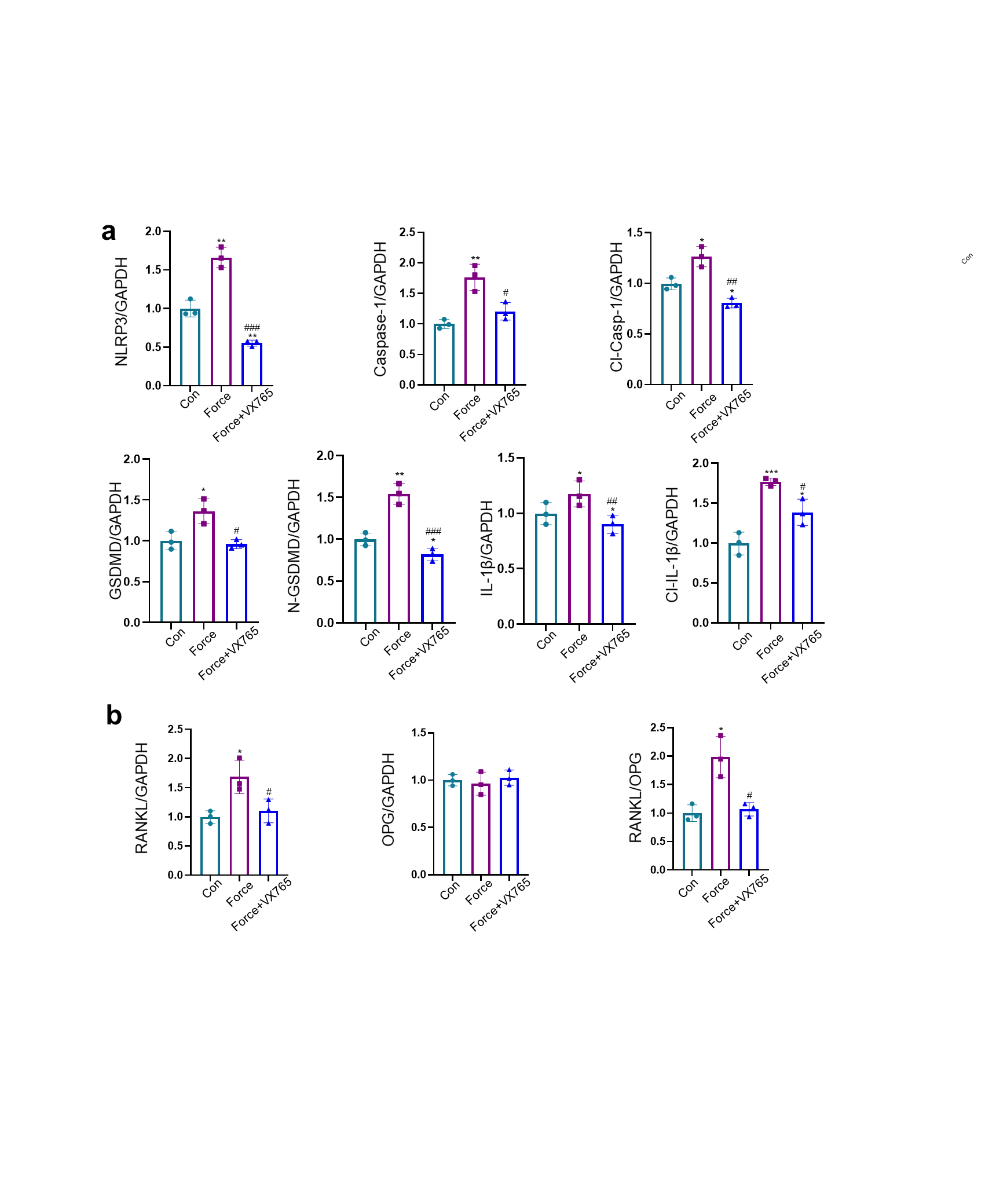


**Figure S6.** **Regulation of Caspase-1 influences RANKL/OPG expression in PDL progenitor cells *in vitro*. a** Semiquantification analysis of Fig. 6**a** (Western blotting of pyroptosis-related proteins in PDL progenitor cells under 1.5 g/cm^2^ force loading for 6 h with or without the appliance of the Caspase-1 inhibitor VX765). **b** Semiquantification analysis of Fig. 6**c** (western blotting of RANKL and OPG in PDL progenitor cells under 1.5g/cm^2^ mechanical force for 6 h with or without the appliance of VX765). **P* < 0.05, ***P* < 0.01, ****P* < 0.001 versus Con; #*P* < 0.05, ##*P* < 0.01, ###*P* < 0.001 versus Force. Results were presented as mean ± SD. *n* = 3 independent experiments.


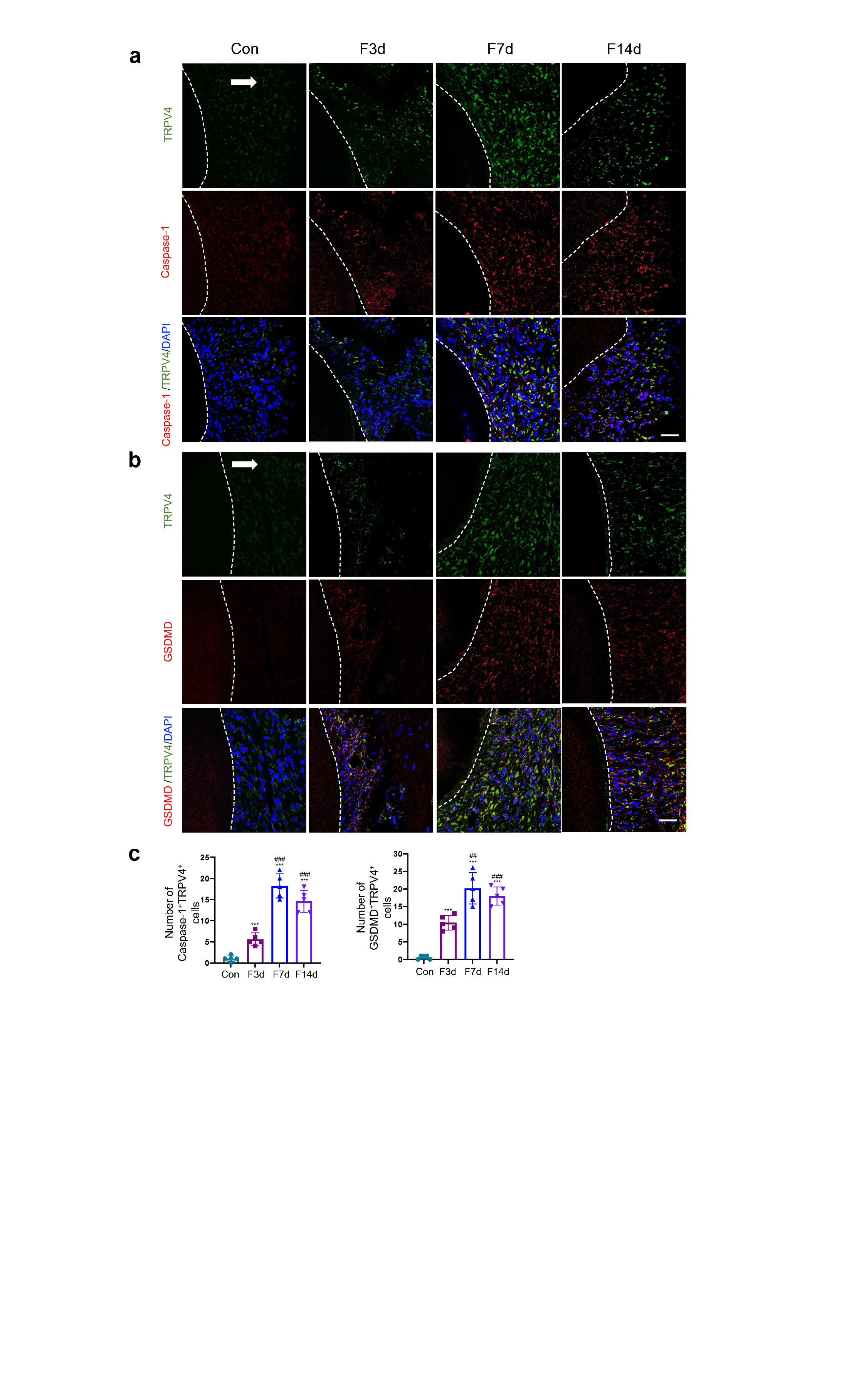


**Figure S7. TRPV4 signaling is involved in force-induced pyroptosis during OTM. a-b** Representative immunofluorescence images of Fig. 7**c**. The number of TRPV4-positive (green) and Caspase-1-positive (red) double-stained cells (merged yellow) as well as TRPV4-positive (green) and GSDMD-positive (red) double-stained cells (merged yellow) increased after force loading for 7 d. Dashed lines mark the outline of distobuccal roots. Arrow represents the direction of the force. Scale bar: 50 µm. **c**  Semiquantification analysis in immunofluorescence images of Fig. 7**c**. ****P* < 0.001 versus Con, ##*P* < 0.01, ###*P* < 0.001 versus F3d.


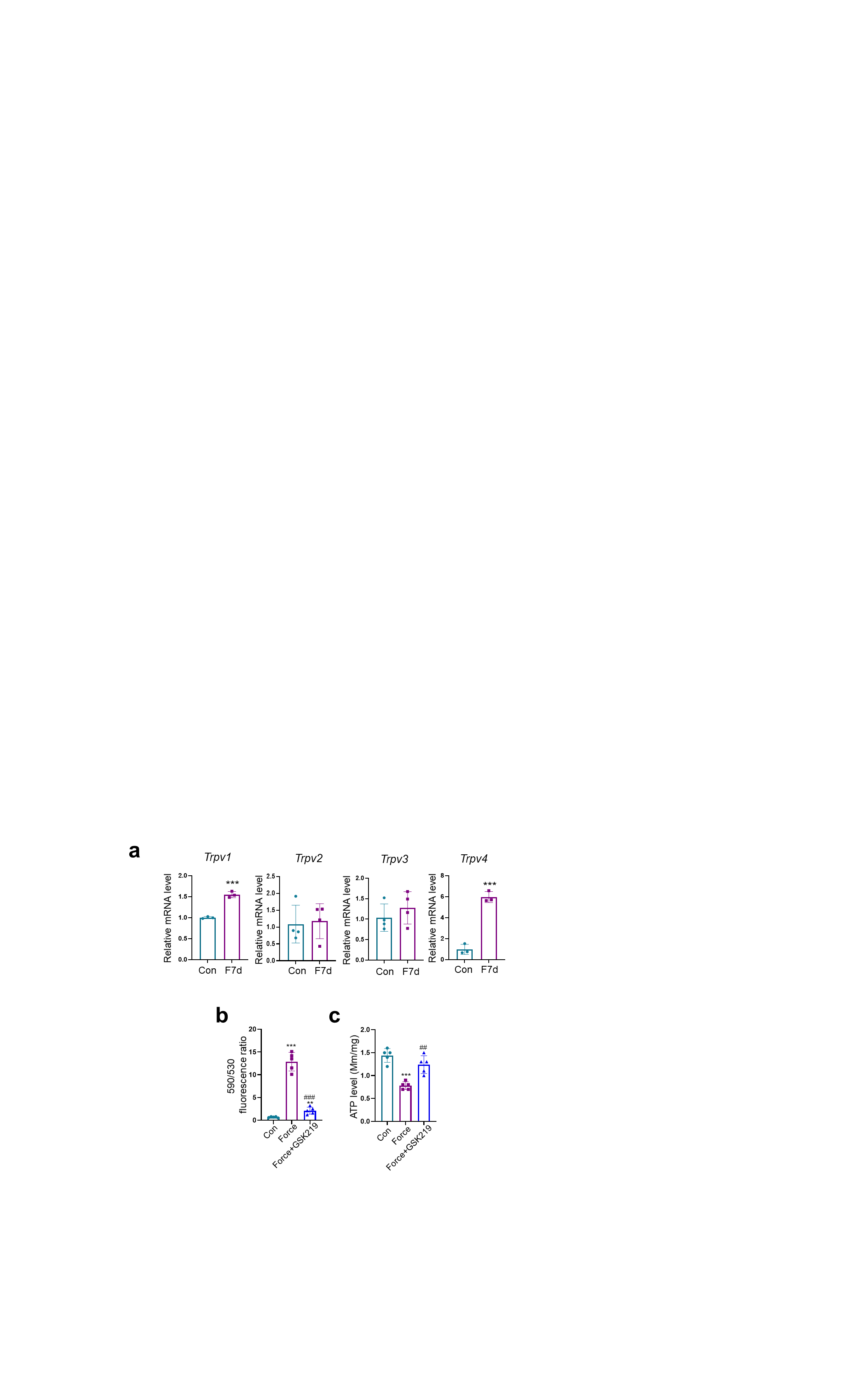


**Figure S8.** **TRPV4 signaling is involved in force-induced pyroptosis in PDL progenitor cells. a** Real time-PCR of *Trpv1*, *Trpv2*, *Trpv3*, and *Trpv4* in periodontal tissues under mechanical force stimuli for 7 d. *n* = 3–4 independent experiments. ****P* < 0.001 versus Con. **b** Statistical results of JC-1 in Fig. 7**e**. 590/530 fluorescence ratio was calculated. *n* = 5 biologically independent samples. ***P* < 0.01, ****P* < 0.001 versus Con, ###*P* < 0.001 versus Force. Results were presented as mean ± SD. **c** The ATP level of PDL progenitor cells. *n* = 5 biologically independent samples. ****P* < 0.001 versus Con, ##*P* < 0.01 versus Force. Results were presented as mean ± SD.

**Supplementary Table 1.** List of reagents or resources used in the study.

| **REAGENT or RESOURCE** | **SOURCE** | **IDENTIFIER** |
| --- | --- | --- |
| **Antibodies** | | |
| Rabbit monoclonal anti - GAPDH | Affinity | Cat# AF7021 |
| Rabbit monoclonal anti - NLRP3 | Thermo Fisher Scientific | Cat# PA5-79740 |
| Rabbit monoclonal anti - Caspase-1 | Affinity | Cat# AF5418 |
| Rabbit monoclonal anti - Cl-Casp-1 | Affinity | Cat# AF4005 |
| Rabbit monoclonal anti - GSDMD/ N-GSDMD | Affinity | Cat# AF4012 |
| Rabbit monoclonal anti - IL-1β | Affinity | Cat# AF5103 |
| Rabbit monoclonal anti - Cl-IL-1β | Affinity | Cat# AF4006 |
| Rabbit monoclonal anti - RANKL | Affinity | Cat# AF0313 |
| Rabbit monoclonal anti - OPG | Affinity | Cat# DF6824 |
| Rabbit monoclonal anti - TRPV4 | Abcam | Cat#[ab39260](http://www.affbiotech.cn/goods-17135-AF4012-GSDMD_Antibody.html) |
| Rabbit monoclonal anti - CD90 | Santa Cruz | Cat#[SC-53456](http://www.affbiotech.cn/goods-17135-AF4012-GSDMD_Antibody.html) |
| Mouse monoclonal anti - CD90 | Abcam | Cat#[ab225](http://www.affbiotech.cn/goods-17135-AF4012-GSDMD_Antibody.html) |
| Mouse monoclonal anti - GSDMD | Santa Cruz | Cat#[SC-393581](http://www.affbiotech.cn/goods-17135-AF4012-GSDMD_Antibody.html) |
| Mouse monoclonal anti - Caspase-1 | Santa Cruz | Cat#[SC-392736](http://www.affbiotech.cn/goods-17135-AF4012-GSDMD_Antibody.html) |
